# Assembly and Characterization of a Pathogen Strain Collection for Produce Safety Applications: Pre-growth Conditions Have a Larger Effect on Peroxyacetic Acid Tolerance Than Strain Diversity

**DOI:** 10.3389/fmicb.2019.01223

**Published:** 2019-05-31

**Authors:** Anna Sophia Harrand, Jasna Kovac, Laura M. Carroll, Veronica Guariglia-Oropeza, David J. Kent, Martin Wiedmann

**Affiliations:** ^1^Department of Food Science, Cornell University, Ithaca, NY, United States; ^2^Department of Food Science, Pennsylvania State University, University Park, PA, United States; ^3^Department of Statistical Science, Cornell University, Ithaca, NY, United States

**Keywords:** whole genome sequencing, produce safety, *Listeria*, *Salmonella*, *E. coli*, peroxyacetic acid

## Abstract

Effective control of foodborne pathogens on produce requires science-based validation of interventions and control strategies, which typically involves challenge studies with a set of bacterial strains representing the target pathogens or appropriate surrogates. In order to facilitate these types of studies, a produce-relevant strain collection was assembled to represent strains from produce outbreaks or pre-harvest environments, including *Listeria monocytogenes* (*n* = 11), *Salmonella enterica* (*n* = 23), shiga-toxin producing *Escherichia coli* (STEC) (*n* = 13), and possible surrogate organisms (*n* = 8); all strains were characterized by whole genome sequencing (WGS). Strain diversity was assured by including the 10 most common *S. enterica* serotypes, *L. monocytogenes* lineages I–IV, and *E. coli* O157 as well as selected “non-O157” STEC serotypes. As it has previously been shown that strains and genetic lineages of a pathogen may differ in their ability to survive different stress conditions, a subset of representative strains for each “pathogen group” (e.g., *Salmonella*, STEC) was selected and assessed for survival of exposure to peroxyacetic acid (PAA) using strains pre-grown under different conditions including (i) low pH, (ii) high salt, (iii) reduced water activity, (iv) different growth phases, (v) minimal medium, and (vi) different temperatures (21°C, 37°C). The results showed that across the three pathogen groups pre-growth conditions had a larger effect on bacterial reduction after PAA exposure as compared to strain diversity. Interestingly, bacteria exposed to salt stress (4.5% NaCl) consistently showed the least reduction after exposure to PAA; however, for STEC, strains pre-grown at 21°C were as tolerant to PAA exposure as strains pre-grown under salt stress. Overall, our data suggests that challenge studies conducted with multi-strain cocktails (pre-grown under a single specific condition) may not necessarily reflect the relevant phenotypic range needed to appropriately assess different intervention strategies.

## Introduction

Control of foodborne pathogens along the supply chain relies on the development and implementation of validated interventions and control strategies, including validated heat treatment schemes or other pathogen reduction steps as well as validated sanitation procedures. While some industries have well established pathogen reduction steps supported by globally recognized validation studies (e.g., pasteurization of milk [Sarkar, 2015]), other food industries and commodities (e.g., baked goods, produce) have an urgent need for scientific validation of different control strategies. Validation is particularly challenging for commodities that represent considerable diversity of production and processing practices, such as it is the case for produce.

In addition, new regulations (such as the US Food Safety Modernization Act [FSMA]) place an increasing emphasis on science-based approaches and scientific validation of control strategies and interventions. Scientifically justifiable strain selection and growth conditions for validation and challenge studies are an essential part of a science-based food safety system. Typically, studies evaluating relevant pathogen interventions or growth and survival of pathogens are conducted using multiple strains, which may be used separately or in cocktails (mixture of multiple strains) (Scott et al., 2005). This approach is used to account for strain diversity and to assure that control strategies are designed to deliver the appropriate or required protection even with more tolerant strains. Pathogen strains used in these types of studies (and in “cocktails”) are typically selected to represent subtypes with a known association with a given product (such as “outbreak strains”), while also considering the phylogenetic diversity of a given target pathogen, which typically is addressed by including multiple distinct serotypes and/or phylogenetic groups. For example, for shiga-toxin producing *Escherichia coli* (STEC), representation of *E. coli* O157 as well as other STEC serotypes commonly associated with *E. coli* infections in the United States (e.g., O26, O45, O103, O111, O121, O145 [Gould et al., 2013]) is often desired; these additional serotypes will be referred to here as “non-O157 STEC” serotypes (in the United States this group is sometimes referred to as the “Big Six non-O157 STEC”).

Importantly, a number of studies have shown that different pathogen strains and genetic lineages can differ considerably in their ability to survive stress conditions; this has been well documented for key pathogens of concern to the produce industry including *L. monocytogenes* (De Jesus and Whiting, 2003; Bergholz et al., 2010; Cheng et al., 2015), *S. enterica* (Li et al., 2012; Andino and Hanning, 2015) and STEC (Oh et al., 2009; Carter et al., 2012; Lee et al., 2012; Wang et al., 2012).

While it has been well established that different strains and genetic lineages of a pathogen may differ in their ability to survive and grow under different stress conditions, the physiological state of bacterial cells and the conditions under which bacteria are pre-grown also have a considerable impact on the ability of foodborne pathogens to survive subsequent stress conditions, including produce-relevant interventions (such as chlorine washes) as specifically documented for *L. monocytogenes* (Bergholz et al., 2012, 2013; Poimenidou et al., 2016), *S. enterica* (Yang et al., 2001; Asakura et al., 2002; Gruzdev et al., 2011; Stackhouse et al., 2012; He et al., 2013) and STEC (Gawande and Griffiths, 2005; Shuai et al., 2017). One of the most well documented examples of the effect of environmental conditions on stress tolerance is the observation that *S. enterica* present in low water activity (a_w_) environments are considerably more tolerant to heat treatment than *S. enterica* present in high water activity environments (He et al., 2013; Peña-Meléndez et al., 2014). For example, *S. enterica* serotype Tennessee (“*Salmonella* Tennessee”) present in peanut butter with an a_w_ of 0.2 showed less than a 3 log reduction after treatment at 90°C for 20 min, while reduction in peanut butter with an a_w_ of 0.8 was around 5 log, a difference of at least 2 log reduction (He et al., 2013). These findings do suggest a need for further studies that evaluate and compare the effects of genetic diversity and growth conditions on subsequent stress tolerance and growth phenotypes. Challenge study guidance documents typically specify that “for either inactivation or growth studies, adaptation of cells should attempt to mimic the likely physiological state of the organism at the time it contaminates the food” (National Advisory Committee on Microbiological Criteria for Foods, 2010). However, there is limited specific guidance or data available that would help to determine which strains or growth conditions should be used for challenge or validation studies if no specific physiological state can easily be defined for contaminating pathogens, as likely in the produce industry where contamination of a single commodity can originate from very different environments.

The aim of this study was to (i) assemble a strain collection including *S. enterica, L. monocytogenes* and STEC, as well as possible surrogate organisms relevant for produce safety (with an initial bias toward United States relevant strains) and to (ii) use a diverse subset of strains from this collection to formally assess the effect of pre-growth conditions on subsequent survival of produce-relevant interventions, using exposure to peroxyacetic acid (PAA) as a model. Since PAA was patented in 1950 to treat fruits and vegetables to reduce spoilage its application for fresh produce has been well established (Greenspan and Margulies, 1950; Wisniewsky et al., 2000; Gonzalez et al., 2004; Hellstrom et al., 2006). PAA is also commonly used in wash water as well as for sanitation of food contact surfaces due to its activity over a broad temperature range and even under presence of organic matter (Lokkesmoe and Olson, 1995; Rodgers et al., 2004). Growth conditions used here were selected to focus on conditions that are either produce relevant (e.g., growth at 21°C) or that have previously been linked to increased stress tolerance, including low water activity (Goepfert et al., 1970; Mattick et al., 2000), acid stress (Lou and Yousef, 1997; Greenacre et al., 2006), salt stress (Begley et al., 2002; Bergholz et al., 2012, 2013), and minimal medium (Jenkins et al., 1988, 1990; Kenyon et al., 2002). The data from this study will help the produce industry to justify the use of (i) specific pathogen and surrogate strains and (ii) specific pre-growth or pre-adaptation conditions for validation studies. In addition, this study will provide access to a well characterized standard strain collection with all strains characterized by whole genome sequencing (WGS).

## Materials and Methods

### Strain Collection Assembly

In order to assemble a produce-relevant strain collection, an initial draft collection was proposed that included strains linked to fresh produce outbreaks and pre-harvest environments, including *S. enterica*, STEC, and *L. monocytogenes* as well as select relevant surrogate strains. As a first step, pathogen strains isolated from human cases or food with key produce-associated outbreaks (e.g., the 2011 listeriosis outbreak linked to consumption of contaminated cantaloupe) were selected. This initial collection was supplemented with non-produce-associated strains as needed to represent the pathogen diversity for a given group. For example, produce-related STEC strains that were initially selected included *E. coli* O157 as well as two non-O157 STEC serotypes (i.e., O121, O26), these strains were supplemented with seven other STEC strains to represent the most common clinically associated serotypes in the United States (i.e., serotype O145, O111, O45 and O103 [Gould et al., 2013]). Surrogate organisms for inclusion in the initial draft strain collection were selected to include strains that had previously been used in produce-relevant validation or challenge studies, e.g., the rifampicin resistant *E. coli* strain TVS 353 (also designated W778), which has been used in a number of studies that assessed survival of *E. coli* on leafy greens (Tomás-Callejas et al., 2011; Weller et al., 2017a; Cai et al., 2018; Wright et al., 2018). The initial draft strain collection included 13 *L. monocytogenes*, 24 *S. enterica*, 10 STEC, as well as 10 surrogate organisms (5 additional *E. coli*, two *Listeria innocua*, one *Listeria marthii*, one *Enterococcus faecalis*, and one *Enterococcus faecium*).

An electronic survey was sent to 30 US-based experts from industry, academia, and government to solicit their input on the initial draft strain collection for its suitability to evaluate produce-relevant pathogens as well as surrogates for different phenotypic characteristics relevant to produce production and distribution (e.g., survival under selected stress conditions and in the presence of sanitizer); see Supplementary Table 1 for this survey, which includes a list of all strains in the draft collection. Experts were asked to rate each strain on a scale of 1 to 5 (1 – strain irrelevant, do not include, 2 – strain may be relevant, 3 – uncertain, strain may or may not be relevant, 4 – important, should be included, 5 – very important, must be included); information provided on the proposed strain collection included ID numbers, isolate origin (e.g., associated outbreak), and references that detailed the strain history or outbreak. Strains were selected for inclusion in the final strain set if at least 50% of respondents considered a strain as important (score of 4 or 5). Reviewers also had the option to suggest inclusion of additional strains; suggested strains were evaluated by the authors, who decided on their inclusion in the final strain set.

### Bacterial Strain Collection Composition and Storage

The final strain collection included (i) 11 *L. monocytogenes*, (ii) 23 *S. enterica*, (iii) 13 STEC, and (iv) 8 surrogate organisms (Table 1). All strains are stored in Brain Heart Infusion (BHI; Difco, Becton Dickinson, Sparks, MD) with 15% glycerol at -80°C.

**Table 1:** Final produce-relevant strains included in the collection *L. monocytogenes, S. enterica*, and *E. coli*, and surrogate organisms.

Serotype (lineage, clonal complex)^a^	FSL ID	Previous ID (provided by)^b^	Isolate source (^∗^ indicates outbreak associated sources)^c^	SRA/Genome^e^	References^c^
***Salmonella enterica***					
Saintpaul^†^	R9-5400	CFSAN004126 (FDA)	Jalepeno peppers, 2008^∗^	SRR5863018/PDBQ00000000	Centers for Disease Control and Prevention [CDC], 2008b
Tennessee	R9-5402	CFSAN001371 (FDA)	Peanut butter, 2006–2007^∗^	SRR5863017/PDBP00000000	Centers for Disease Control and Prevention [CDC], 2007
Typhimurium	R9-5494	K2442 (CDC)	Orange juice, 2005^∗^	SRR5863016/PDBO00000000	Jain et al., 2009
Typhimurium	R9-5409	CFSAN016159 (FDA)	Peanut butter, 2008–2009^∗^	SRR5863015/PDBN00000000	Centers for Disease Control and Prevention [CDC], 2009
Poona	R9-6568	PTVS001 (UCDavis)	Cantaloupe, 2000–2002^∗^	SRR5863022/PDBM00000000	Centers for Disease Control and Prevention [CDC], 2002a
Poona – Rif^R^	R9-6569	PTVS026 (UCDavis)	Derivative of PTSV001	SRR5863021/PDBL00000000	–
Enteritidis – PT30^†^	R9-5272	ATCC BAA 1045	Almonds, 2000–2001	SRR5863020/PDBK00000000	Isaacs et al., 2005
Javiana	R9-5273	ATCC BAA-1593	Tomatoes, 2002^∗^	SRR5863019/PDBJ00000000	Srikantiah et al., 2005
Newport-Rif^R^	R9-5251	MDD314R (UoFlorida)	Derivative of MDD314	SRR5863014/PDBI00000000	–
Newport – antimicrobial susceptible	R9-5252	MDD314 (UoFlorida)	Tomatoes, 2002 and 2005^∗^	SRR5863013/PDBH00000000^g^	Greene et al., 2008
Senftenberg 775W	R9-5274	ATCC 43845	Chinese egg powder, 1941^∗^	SRR5863026/PDBG00000000	Winter et al., 1946
Heidelberg	R9-5495	2012K-1421 (CDC)	Poultry facility, 2012–2013	SRR1616738^f^	
I 4,[5],12:i:–	R9-5496	2011K-0033 (CDC)	Alfalfa sprouts, 2010–2011^∗^	SRR5863025/PDBF00000000	Centers for Disease Control and Prevention [CDC], 2011a
Litchfield^†^	R9-5344	BAC0800000628 (NYSDOH)	Cantaloupe, 2008^∗^	SRR5863028/PDBE00000000	Centers for Disease Control and Prevention [CDC], 2008a
Poona^†^	R9-5502	2015K-0961 (CDC)	Cucumber 2015^∗^	SRR5863027/PDBD0000000	Centers for Disease Control and Prevention [CDC], 2015b
Anatum	R9-5219	LJH#720 (UCDavis)	Raw almonds	SRR5863030/PDBC00000000^g^	Danyluk et al., 2007
Anatum – Nal^R^	R9-5220	LJH#1217 (UCDavis)	Derivative of LJH#720	SRR5863029/PDBB00000000^g^	–
Infantis	R9-5497	2012K-1623 (CDC)	Dry Pet food 2012	SRR5863032/PDBA00000000	Centers for Disease Control and Prevention [CDC], 2012b
Muenchen	R9-5498	2016K-0150 (CDC)	Alfalfa sprouts, 2016^∗^	SRR5863031/PDAZ00000000	Centers for Disease Control and Prevention [CDC], 2016a
I 13,23:b: –	R9-5499	2011K-1002 (CDC)	–	SRR5863024/PDAY00000000	–
Newport-MDR	R9-5504	AM12179 (CDC)	Undercooked ground beef, 2002	SRR5863023/PDAX00000000	Centers for Disease Control and Prevention [CDC], 2002b
Montevideo	R9-5406	531954 (FDA)	–	SRR5863049/PDAW00000000	–
Enteriditis	R9-5505	2015K-0277 (CDC)	Bean sprouts, 2014^∗^	SRR5863050/PDAV00000000	Centers for Disease Control and Prevention [CDC], 2015a
***Listeria monocytogenes***					
4b (I, CC1)	J1-0108	TS27/L4738/DD6304 (ILSI)	Coleslaw, 1981^∗^	SRR5863034/PDAL00000000^g^	Fugett et al., 2006
1/2b (I, CC3)	R2-0503	G6054 (ILSI)	Human, 1994	SRR5863033/PDAK00000000	Fugett et al., 2006
4d (I, CC1)	J1-0107	TS26/L4742/DD6303 (ILSI)	Coleslaw, 1981^∗^	SRR5863040/PDAJ00000000^g^	Nightingale et al., 2007
1/2a (II, CC11)	J1-0101	G3975/DD6292/F6900 (ILSI)	Hot dog^∗^	SRR5863039/PDAI00000000^g^	Centers for Disease Control and Prevention [CDC], 1989
1/2a (II, CC29) ^†^	R9-0506	L2625 (CDC)	Cantaloupe, 2011^∗^	SRR5863038/PDAH00000000^g^	Centers for Disease Control and Prevention [CDC], 2011b
1/2 b (I, CC88) ^†^	R9-5411	897760 (FDA)	Caramel apple, 2015^d^	SRR1812790^f^	–
4b (I, ST382) ^†^	R9-5506	PNUSAL001751 (CDC)	Packaged salad, 2016^∗^	SRR2485319^f^	Burall et al., 2017
4b (I, CC554)	R9-5507	PNUSAL000954 (CDC)	Sprouts, 2014^∗^	SRR1562154^f^	Centers for Disease Control and Prevention [CDC], 2015c
4a (II, CC396) ^†^	J1-0031	LM36 (ILSI)	Human	SRR5863041/PDAE00000000^g^	Fugett et al., 2006
4b (IV, ST382)	J1-0158	(ILSI)	Goat, 1997	SRR5863011/PDAD00000000^g^	Fugett et al., 2006
1/2a (II, ST364)	S10-2161	–	Soil spinach field	SRR5863012/PNRM00000000^g^	Weller et al., 2015
***Escherichia coli***					
O121:H19	R9-5509	2014C-3598 (CDC)	Raw clover sprouts, 2014^∗^	SRR5863051/PDAU00000000	Centers for Disease Control and Prevention [CDC], 2014
O104:H4	R9-5256	2011C-3493 (USDA)	Sprouts, Germany, 2011^∗^	–/CP003289.1^f^	Ahmed et al., 2012
O104:H4	R9-5257	2009EL-2071 (USDA)	Human, Republic of Georgia, 2009	SRR5863052/PDAT00000000	Ahmed et al., 2012
O104:H4^†^	R9-5258	2009EL-2050 (USDA)	Human, Republic of Georgia, 2009	SRR5863045/PDAS00000000	Ahmed et al., 2012
O26:H11	R9-5512	2012C-4704 (CDC)	Raw clover sprouts, 2012^∗^	SRR5863046/PDAR00000000	Centers for Disease Control and Prevention [CDC], 2012a
O157:H7^†^	R9-5271	RM6012 (Wisconsin State Lab)	Baby spinach, 2006^∗^	SRR5863047/PDAQ00000000^g^	Centers for Disease Control and Prevention [CDC], 2006
O157:H7	R9-5513	2016C-3325 (CDC)	Alfalfa sprouts, 2016^∗^	SRR5863048/PDAP00000000	Centers for Disease Control and Prevention [CDC], 2016b
O111:H8	R9-5345	(Cornell)	Apple cider	SRR5863043/PNRN00000000^g^	–
O111:H8^†^	R9-5515	2014C-3989 (CDC)	Cabbage salad, 2014^∗^	SRR5863044/PDAO00000000	–
O145:NM^†^	R9-5516	2010C-3510 (CDC)	Shredded romaine lettuce, 2010^∗^	–/GCA_000615175.1^f^	Centers for Disease Control and Prevention [CDC], 2010
O103:H2^†^	R9-5517	2015C-5140 (CDC)	Human	SRR5863036/PDAN00000000	–
O26:H11	R9-5639	TW016501 (STEC Center)	Sprout, 2012^∗^	SRR5863035/PDAM00000000	–
O45:H2	R9-6071	CSU E1-134 (Texas Tech)	–	SRR5863006/PCZZ00000000^g^	–
***Surrogate organisms***					
*L. innocua*^†^	C2-0008	–	Fish processing plant, 2000	SRR5863009/PNRL00000000^g^	–
*E. coli* O88:H25^†^	R9-4077	TVS 353 (UCDavis)	Generic *E. coli*, irrigation water	SRR5863010/PDAC00000000^g^	(Tomás-Callejas et al., 2011)
*E. coli* O88:H25	R9-4078	TVS 354 (UCDavis)	Generic *E. coli*, romaine lettuce	SRR5863007/PDAB00000000^g^	(Tomás-Callejas et al., 2011)
*E. coli* O88:H25	R9-4079	TVS 355 (UCDavis)	Generic *E. coli*, soil	SRR5863008/PDAA00000000^g^	(Tomás-Callejas et al., 2011)
*E. coli* O157:H7	R9-3467	ATCC 700728	Naturally occurring non-pathogenic *E. coli*	–/GCA_000335055.2^e^	(Atwill et al., 2015)
*Salmonella* Typhimurium	R9-6231	MHM108 (UoFlorida)	Avirulent Salmonella	–	(de Moraes et al., 2016)
*Salmonella* Typhimurium^†^	R9-6232	MHM112 (UoFlorida)	Avirulent Salmonella	–/LONA00000000.1^e^	(de Moraes et al., 2016)
*E. faecium*^†^	R9-5275	ATCC 8459	Salmonella Surrogate (*E. faecium*)	–/CP004063.1^e^	(Kopit et al., 2014)


*^*a*^Antibiotic resistant strains are designated with Rif^*R*^ (rifampicin resistance), and Nal^*R*^ (nalidixic acid resistance); ^†^indicates isolates selected for phenotypic experiments. ^*b*^Abbreviations for isolate sources include FDA (Food and Drug Administration); CDC (Centers for Disease Control and Prevention); UCDavis (University California Davis); American Type Culture Collection (ATCC), UoFlorida (University of Florida); NYSDOH (New York State Department of Health); Cornell (Cornell University); International Life Sciences institute (ILSI), STEC Center (STEC Center at Michigan State University); Texas Tech (Texas Tech University). ^*c*^“–” Indicates that information is not available. ^*d*^This isolate was classified as multilocus sequence type (ST) 88 (the predicted serotype for this ST is 1/2b), isolates associated with the 2014–2015 caramel apple outbreak were designated ST1 and 382 (Angelo et al., 2017), suggesting that this isolate may not be associated with the caramel apple outbreak, even though it was isolated from caramel apples. ^*e*^First number indicates the SRA accession number and second number indicates the genome accession number; “–” indicates that a given number is not available. All isolates marked with “f,” sequencing data were retrieved from NCBI, all isolates marked with “g,” were sequenced on the HiSeq 2500 platform; all other isolates were sequenced on the MiSeq platform.*

### Library Preparation and Whole Genome Sequencing

Isolates were streaked from glycerol stocks onto BHI agar plates and plates were incubated at 37°C for 24 h. An overnight culture was prepared by inoculating 5 mL BHI broth with a single colony, followed by incubation at 37°C for 12–14 h. Following manufacturer’s instructions for DNA extraction, 2 mL of the 12–14 h culture was pelleted and used for DNA extraction (DNeasy Blood and Tissue kit, Qiagen, Valencia, CA). Gram-positive bacteria were pre-treated in 200 μL lysis solution (20 mg/mL lysozyme, 20 mM Tris–HCl, 2 mM EDTA, 1.2% Triton X-100). DNA was eluted in 50 μL of 10 mM Tris–HCl at pH 7.5, followed by spectrophotometric assessment of DNA purity with a NanoDrop 2000 (Thermo Fisher Scientific, Waltham, MA) and DNA quantification with a fluorescent nucleic acid dye (Qubit dsDNA HS Assay Kit, Thermo Fisher Scientific) and a Qubit 2.0 fluorometer (Thermo Fisher Scientific). Libraries were prepared for sequencing with Nextera XT DNA sample preparation Kit and the associated Nextera XT Index Kit with 96 indices (Illumina, Inc., San Diego, CA). Library preparation was conducted according to the PulseNet standard operation procedure “Laboratory Standard Operating Procedure for Pulsenet Nextera XT Library Prep and Run Setup for the Illumina Miseq”^1^. Pooled samples were sequenced on an Illumina MiSeq platform with 2× 250 bp paired-end reads (Animal Health Diagnostic Center Cornell University) or HiSeq 2500 rapid run with 2× 100 bp paired-end reads (Genomics Facility of Cornell University).

### Genome Assembly and Analyses

Adapters were removed from sequences using Trimmomatic v 0.33 (Bolger et al., 2014) followed by quality assessment using FastQC v 0.11.4^2^. Sequences were assembled *de novo* with SPAdes version 3.8.0 (Bankevich et al., 2012). Quality control of assemblies was performed with QUAST v 3.2 (Gurevich et al., 2013) and average coverage determined using SAMtools v 1.4.1 (Li et al., 2009). Contigs smaller than 200 bp were removed and the remaining contigs were searched against Kraken (Wood and Salzberg, 2014), using BLAST, to confirm strain identity. Additionally, serotypes were confirmed with Seqsero^3^ for *S. enterica* (Zhang et al., 2015) and SeroTypeFinder for *E. coli* (Joensen et al., 2015). A standard set of 21 sequenced *Listeria* genomes previously described by Liao et al. (2017) was used in a SNP-based phylogenetic analysis to confirm lineages for *Listeria* strains (Liao et al., 2017). *S. enterica* with acquired antibiotic resistance (e.g., rifampicin, naldixic acid resistance) were compared to their wildtype parent to identify high quality SNPs using the CFSAN SNP Pipeline (Davis et al., 2015). BLASTX was used to identify genes if SNPs were located in potential open reading frames (ORFs) (States and Gish, 1994).

The core SNP based analysis was performed using kSNP v 3, with estimated optimal kmer size 13 (determined using Kchooser), for each bacterial group including (i) *Listeria*, (ii) *S. enterica*, and (iii) *E. coli* (Gardner et al., 2015). A maximum-likelihood phylogeny based on core genome SNP was generated with 1000 bootstrap repetitions in RAxML for each bacterial group. Surrogate strain sequences were integrated into phylogenetic analysis with their associated bacterial group except for *E. faecium* (ATCC 8459) (Stamatakis, 2015). Phylogenetic trees were edited using FigTree v 1.3.4^4^.

As an example, for additional screening for mutations, the selected strains were analyzed for mutations in key stress response regulator genes, including *sigB* (for *Listeria*) and *rpoS* (for *S. enterica* and *E. coli*). Reference genes used were *sigB* of EGD-e (259 aa; NCBI accession no. NC_003210.1:930671-931450), *rpoS* of *S. enterica* Typhi str. CT18 (330 aa, NCBI accession no. NC_003198.1:c2916069-2915077) and *rpoS* of *E. coli* K-12 (330 aa; NC_000913.3:c2867551-2866559); the K12 reference sequence was selected to represent a strain that was lacking any *rpoS* mutations that had been reported for some K12 strains, including the codon 33 amber mutation (Subbarayan and Sarkar, 2004). To identify non-synonymous mutations or premature stop codons, amino acid sequences were aligned using MUSCLE (Edgar, 2004).

### Pre-growth Conditions Before PAA Exposure

Bacterial isolates were streaked from glycerol stocks onto Tryptic Soy Agar plates (TSA; Difco, Becton Dickinson, Sparks, MD) and incubated at 37°C for 24 h; following incubation plates were held at 4°C for a minimum of 24 h and a maximum of 7 days. Single colonies from these plates were inoculated into 5 mL of TSB, followed by incubation at 37°C with shaking for 12–14 h. These cultures were used to inoculate, at a 1:1000 dilution, pre-warmed side-arm flasks (Nephelo Flasks/C38 300 mL; Belco, Vineland, NJ) that contained either 30 or 100 mL of growth medium. Bacterial cultures were grown without shaking to early stationary phase at 37°C except when the pre-growth condition was defined as “mid-log phase” or “21°C.” Growth curves were generated for each strain in each condition to determine the OD for each growth phase using a spectrophotometer (20D+, Thermo Fisher Scientific) with a linear range from 0.2 to 0.7. Appropriate dilutions were made prior to reading if necessary, to stay within the linear range. For this study, seven different pre-growth conditions were selected, including (i) low pH (pH 5.0 for *S. enterica* and *E. coli*; pH 5.5 for *Listeria*), (ii) high salt (4.5% NaCl), (iii) reduced water activity (0.96 for *S. enterica* and *E. coli*; 0.95 for *Listeria*), (iv) two different growth phases (mid-log phase, stationary phase), (v) minimal medium including M9, prepared as previously described (Green and Sambrook, 2012) using 0.1% (w/v) casamino acids and either 0.4% glucose (w/v) (for *S. enterica*) or 0.3% fructose (w/v) (*E. coli*) and chemically defined minimal medium with 10 mM glucose for *Listeria* (Premaratne et al., 1991; Peña-Meléndez et al., 2014) and (vi) two different temperatures (37°C, 21°C). Water activity was adjusted using glycerol at 15.6% (v/v) and 13% (v/v) to achieve an a_w_ of 0.95 (*Listeria*) and 0.96 (*S. enterica* and *E. coli*), respectively. The pH was adjusted using lactic acid and high salt environment was generated with additional 4% NaCl (w/v). The parameters for pre-growth were chosen based on preliminary experiments that identified the most stressful conditions (e.g., lowest pH) that would still allow for reproducible growth curves. The pre-growth conditions chosen for this project are only a selection of possible relevant stress conditions. For examples, pathogen contamination can occur both at the preharvest stages (e.g., from soil, wildlife feces) or post-harvest environments (e.g., dry or wet processing plant environments); hence pre-growth conditions may reflect a wide range of environmental conditions. Importantly, all stresses selected for this study have been previously shown to increase stress tolerance including low water activity (Goepfert et al., 1970; Mattick et al., 2000), acid stress (Lou and Yousef, 1997; Greenacre et al., 2006), salt stress (Begley et al., 2002; Bergholz et al., 2012, 2013), and minimal medium (Jenkins et al., 1988, 1990; Kenyon et al., 2002). Further details on all pre-growth conditions are reported in Supplementary Table 2 and Supplementary Figures 1–3.

### PAA Treatment

Phylogenetic and SNP data were used to select four diverse wild type strains for *L. monocytogenes* and *S. enterica* as well as five diverse wild type strains for STEC for phenotypic characterization. These strains were supplemented with one surrogate for *L. monocytogenes* (*L. innocua* FSL C2-0008), two surrogates for *S. enterica* (the avirulent *S. enterica* MHM112 and *E. faecium* ATCC 8459), and one surrogate for STEC (*E. coli* TVS 353). Bacterial cultures pre-grown under different pre-growth conditions were exposed to PAA (Tsunami, Ecolab, St. Paul, MN) and PAA concentration was measured using Reflectoquant (RQflex 10, Millipore, Darmstadt, Germany). For each treatment, 1 mL of bacterial culture was added to 9 mL PAA (in a 15 mL Falcon tube) for a final concentration of either 60 ppm (*Listeria*), or 40 ppm (*E. coli, S. enterica*) followed by mixing through four inversions and incubation for 45 s (the 45 s time period included the time required for the four inversions). The sanitizer solution was inactivated by adding 100 μL 50% Na_2_S_2_O_3_ (w/v) immediately after the 45 s incubation, followed by four inversions of the tube to assure complete mixing; the four inversions took about 10 s and were completed after the 45 s exposure time. Control cultures were treated with phosphate-buffer saline solution (PBS) instead of PAA. Immediately after sanitizer inactivation, 50 μL of the appropriate dilutions were plated in duplicates on TSA plates using a spiral plater (Autoplate 4000, Advanced Instruments Inc., Norwood, MA). Plates were incubated at 37°C for 24 h (*S. enterica, E. coli*) or 48 h (*Listeria*). Colonies were enumerated using Color Q-Count (Model 530, Advanced Instruments Inc.). The PAA concentrations chosen here are below the maximum use level concentration of 80 ppm for wash water detailed in the US Code of Federal Regulations Title 21 (21CFR173.315); PAA concentrations were chosen to typically yield bacterial numbers above the detection limit of 100 CFU/mL after PAA treatment (in order to allow for quantification of die-off).

### Statistical Analyses

Statistical analyses were performed in R (version 3.1, R Core Team, Vienna, Austria). A generalized linear model was fitted to the binomial proportion of surviving cells with a log_e_ link function using lme4 package (Bates et al., 2015); independent variables were the crossed random effects of strain and condition. The arithmetic mean of observed log reduction was reported when survival of surrogate organisms was compared to a particular strain set and limit of detection was substituted for values where post-sanitizer count was zero. All experiments were conducted in biological triplicate.

### WWW-Based Data Access

Trimmed raw reads and assembled genomes for all strains were submitted under the BioProject ID PRJNA395587 to NCBI’s Sequence Read Archive (SRA) and GenBank (Table 1). Information about the strain collection is also available at https://foodsafety.foodscience.cornell.edu/research-and-publications/-strain-collection. Data associated with each strain (e.g., source isolation, serotype, published papers associated with a given strain) are also available on Food Microbe Tracker^5^ (Vangay et al., 2013).

### Strain Availability

Strain requests within the United States or internationally can be directed to the original provider or the Food Safety Laboratory (FSL; Department of Food Science, Cornell University, Ithaca, NY; e-mail: mw16@cornell.edu). Strain requests for strains provided by the FDA, USDA, ATCC or the STEC Center have to be directed to these institutions (see Supplementary Table 3 for current contact information).

## Results

### Assembly of Final Collection

Among the 30 experts that were surveyed for their feedback on an initial draft collection that included a total of 57 strains, 19 provided responses including experts in academia (*n* = 6), government (*n* = 5), and industry (*n* = 8), all with at least 10 years of experience in food safety. More than 50% of experts who responded classified 33 of the 57 strains as “important” (ranking of 4 or 5); all of these strains were included in the final set. While 24 strains were classified as important by <50% of experts, seven of them were still included in the final set as their inclusion was necessary to assure strain diversity including the 10 most common *S. enterica* serotypes in the US-based on the incidence rate of *Salmonella* infections (Foodborne Diseases Active Surveillance and Network, 2017), all four *L. monocytogenes* lineages, and the most common non-O157 STEC serotypes linked to human illnesses in the United States (Gould et al., 2013). For example, the *L. monocytogenes* lineage IV strain FSL J1-158 (isolated from a goat) was classified as important by only 7/19 experts but was included to assure presence of at least one lineage IV strain in the final strain set. Expert reviewers also suggested 10 additional strains for inclusion. Of the suggested strains, four were added to the final strain collection. In addition, four parent strains were acquired along with antibiotic resistant derivatives of the parent strains, e.g., *Salmonella* Poona FSL R9-6569 is the rifampicin resistant derivative of FSL R9-6568. During the strain acquisition process and after completion of the survey, an additional seven strains were submitted to the strain collection by experts in academia and government e.g., the avirulent *Salmonella* Typhimurium strain MHM112 (FSL R9-6232) (de Moraes et al., 2016). The final collection includes a total of 55 strains, which are discussed in more detail below. While all strains were assigned Food Safety Lab (“FSL”) numbers, in this publication, the previous ID numbers will be used for previously reported surrogates to be consistent with previously published literature.

Strains for the collection were obtained from either (i) previously described collections (e.g., ILSI NA Listeria strain collection [Fugett et al., 2006], ATCC [Clark and Geary, 1974]), the Cornell Food Safety Lab (FSL) collection or (ii) various outside sources, e.g., Food and Drug Administration (FDA), Centers for Disease Control and Prevention (CDC), United States Department of Agriculture (USDA), Wisconsin State Laboratory of Hygiene, Texas Tech University, STEC Center Michigan State University, University of California Davis, and University of Florida (Table 1).

### *Listeria monocytogenes* Strain Set

The *L. monocytogenes* strain set is comprised of 11 strains, 4 of them from listeriosis cases linked to produce, including cantaloupe (2011), packaged salad (2016) and sprouts (2014); one strain was obtained from soil collected in a spinach field (Weller et al., 2015). An additional six strains were included to ensure inclusion of lineages I–IV. The final strain set represented four *Listeria* serotypes, as well as six lineage I strains, three lineage II strains, and one strain for each lineage III and lineage IV. In addition to the 11 *Listeria* detailed above, the strain set includes *L. innocua* as a possible surrogate organism for *L. monocytogenes* (Girardin et al., 2005; Friedly et al., 2008; Omac et al., 2015); we specifically included *L. innocua* strain FSL C2-0008 (Table 1).

**FIGURE 1:**
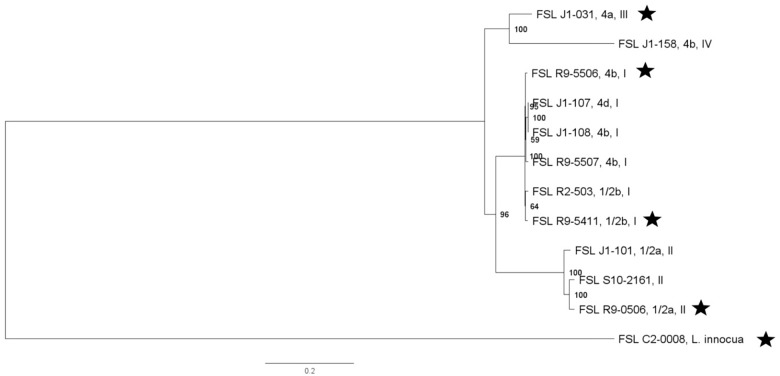
*Listeria* maximum-likelihood tree based on core SNP analysis using kSNP. The phylogeny for strains was inferred using RAxML and tree was rooted by midpoint. The strain FSL ID is followed by serotype and lineage. The node labels represent bootstrap values of 1000 repetitions. The bar indicates 0.2 substitutions per site. Strains marked with a star were selected as representative strains for phenotypic analyses (i.e., PAA experiments).

For three of the *Listeria* strains, WGS data were already available. WGS data for all other strains were generated (see Supplementary Table 4 for detailed WGS data). A core SNP maximum-likelihood tree showed that strains clustered by their lineages (Figure 1). Among all *Listeria*, the number of pairwise core SNP differences ranged from 1 to 6,014 SNPs. The maximum number of core SNP differences within lineage I strains was 359 SNPs as compared to 1,055 SNPs within lineage II. Lineage I isolates FSL J1-0107 and FSL J1-0108, showed no SNP differences based on the kSNP-based core SNPs, consistent with the fact that both isolates were obtained from the same outbreak. In contrast, high quality SNP analysis identified 14 SNP differences between these two isolates. Also, all strains were assessed for potential mutations in *sigB*, which encodes the alternative sigma factor σ^B^; mutations in this gene may reduce stress tolerance (Raengpradub et al., 2008). Among the 12 strains, SigB was highly conserved with only four polymorphic amino acid sites (see Supplementary Figure 4 for details).

### *Salmonella enterica* Strain Set

The *S. enterica* strain set is comprised of 23 strains; 13 of these strains are from outbreaks linked to produce, including cantaloupe (two outbreaks, 2000–2002 and 2008), tomatoes (two outbreaks, 2002 and 2005), orange juice (2005), jalapeno peppers (2008), cucumbers (2015), alfalfa sprouts (2016), and bean sprouts (2014). Three strains were associated with outbreaks linked to almonds (2000–2001) and peanut butter (two outbreaks, 2006–2007 and 2008–2009). An additional four strains were included to ensure representation of the 10 most common serotypes associated with human illnesses in the United States (Foodborne Diseases Active Surveillance and Network, 2017); the final set represents 16 *S. enterica* serotypes. Finally, the strain set includes two *S. enterica* strains that have previously been used in produce-relevant validation studies (e.g., *Salmonella* Senftenberg FSL R9-5274). In addition to the 23 *S. enterica* detailed above, the strain set also includes three surrogate organisms relevant for *S. enterica*, specifically *E. faecium* ATCC 8459 (FSL R9-5275) and the two avirulent *S.* Typhimurium MHM112 and MHM108 (FSL R9-6232 and FSL R9-6231, respectively).

For one of the 23 *S. enterica* strains (as well as for *E. faecium* ATCC 8459 and the avirulent *Salmonella* Typhimurium MHM112), WGS data were already available; WGS data were generated for all other strains (Table 1). Initial analysis of the WGS data identified two *S. enterica* isolates where the serotype predicted based on WGS did not match the reported serotype (i.e., the serotype reported for the two outbreaks these isolates were associated with). For example, a *Salmonella* Poona isolate (received as representing an isolate from the cantaloupe outbreak in 2000–2002) was identified as serotype Agona based on WGS data, indicating that the wrong isolate was sequenced. These two isolates were subsequently acquired from another source; WGS of these new isolates confirmed that they represented the correct serotype.

**FIGURE 2:**
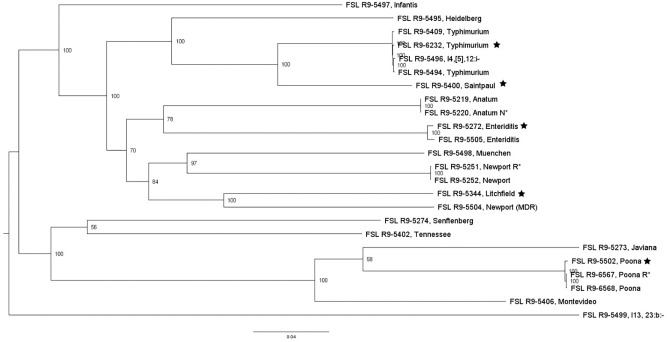
*Salmonella* maximum-likelihood tree based on core SNP analysis using kSNP. The phylogeny for strains was inferred using RAxML and tree was rooted by midpoint. The strain FSL ID is followed by serotype; R^∗^ and N^∗^ indicate rifampicin and nalidixic acid resistant strains, respectively. The node labels represent bootstrap values of 1000 repetitions. The bar indicates 0.04 substitutions per site. Strains marked with a star were selected as representative strains for phenotypic analyses (i.e., PAA experiments).

Whole genome sequencing data for all *S. enterica* strains yielded genome sizes from 4.6 to 5.3 Mbp (see Supplementary Table 4 for detailed WGS data). Among all *S. enterica* strains (excluding antibiotic resistant strains derived from a given parent strain), the number of pairwise core SNP differences ranged from 51 to 13,436 SNPs. A core SNP maximum-likelihood tree showed that strains clustered by serotypes except for (i) the one serotype 4,[5],12:i:- strain, which, as expected, clustered closely within Typhimurium and (ii) one Newport strain, which clustered closely with serotype Litchfield, consistent with previous data that Newport is polyphyletic and represents multiple lineages (Timme et al., 2013; Toboldt et al., 2013; Zheng et al., 2017) (Figure 2).

Further WGS analyses were performed to identify specific mutations in (i) the two *S. enterica* strains that had been selected for resistance to rifampicin (FSL R9-6567, FSL R9-5251) and (ii) one *S. enterica* strain that had been selected for resistance to nalidixic acid (FSL R9-5220). High quality SNP analysis identified a single point mutation in the RNA polymerase subunit B gene (*rpoB*) in the rifampicin resistant strain FSL R9-6567, as compared to its parent strain (FSL R9-6568). The rifampicin resistant strain FSL R9-6567 also had a non-synonymous mutation in *flhE*, which is part of the *flhBAE* operon and has a potential role as a chaperone and contributes to flagellar biosynthesis (Lee et al., 2015). The rifampicin resistant strain FSL R9-5251 showed 9 non-synonymous mutations relative to its parent strain, including two in *rpoC*, which encodes the RNA polymerase β′ subunit, as well as one each in *fadJ* (fatty acid oxidation complex subunit alpha) (Iram and Cronan, 2006), *fadR* (the transcriptional regulator for fatty acid degradation), *hemL* (the glutamate-a-semialdehyde aminotransferase*), glpR* (transcriptional repressor of sugar metabolism), and *yhdA* (encoding the regulatory protein CsrD, which controls degradation of CsrB and CsrC RNA) (Suzuki et al., 2006). The nalidixic resistant strain FSL R9-5220 showed one non-synonymous mutation in *gyrA*, which encodes the DNA gyrase subunit A and plays an essential role in DNA replication (Piddock et al., 1998) (see Supplementary Table 5 for details).

All strains were also assessed for potential mutations in *rpoS*, which encodes the alternative sigma factor (RpoS); mutations in this gene have previously been shown to accumulate during lab passages and may reduce stress tolerance (Sutton et al., 2000). Among the 22 strains, RpoS was highly conserved with only three polymorphic amino acid sites; strain FSL R9-5505 carried a point mutation, which leads to a frameshift and premature stop codon (see Supplementary Figure 5 for details). The surrogate organism (the avirulent *S.* Typhimurium, MHM112) was excluded from the analysis due to low coverage.

### *Escherichia coli* Strain Set

The STEC set is comprised of 13 strains; eight of these strains are from *E. coli* outbreaks linked to produce, including baby spinach (2006), shredded romaine lettuce (2010), sprouts (two outbreaks, 2011 and 2012), clover sprouts (two outbreaks, 2012 and 2014), cabbage salad (2014), and alfalfa sprouts (2016). An additional two strains collected in the Republic of Georgia (2009) and one strain from an outbreak linked to apple cider were also included. In order to ensure inclusion of the non-O157 STEC serotypes, an additional two strains were included representing serotypes O45 and O103. The strain set also includes four surrogate organisms relevant for *E. coli*, including three generic *E. coli* isolated from the environment (FSL R9-4077, FSL R9-4078, FSL R9-4079), as well as a naturally occurring non-pathogenic *E. coli* O157:H7 (FSL R9-3467), all of which have been used previously as surrogates (Tomás-Callejas et al., 2011; Atwill et al., 2015; Weller et al., 2017a,b; Cai et al., 2018; Wright et al., 2018) (Table 1).

**FIGURE 3:**
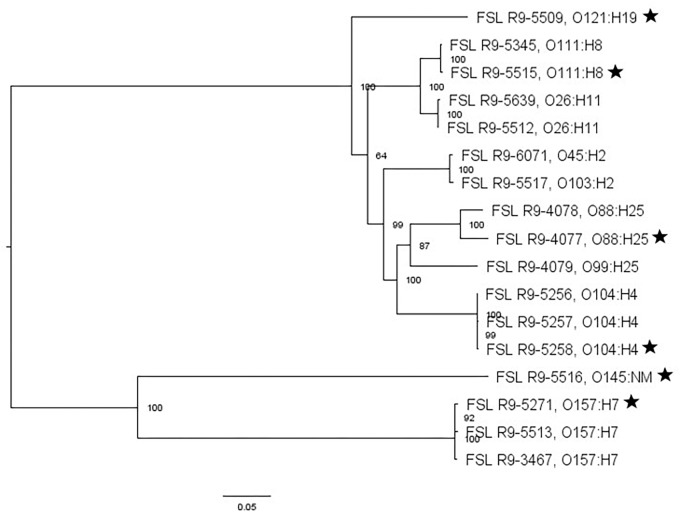
*Escherichia coli* maximum-likelihood tree based on core SNP analysis using kSNP. The phylogeny for strains was inferred using RAxML and tree was rooted by midpoint. The strain FSL ID is followed by serotype. The node labels represent bootstrap values of 1000 repetitions. The bar indicates 0.05 substitutions per site. Strains marked with a star were selected as representative strains for phenotypic analyses (i.e., PAA experiments).

For two of the 13 STEC strains (as well as the non-pathogenic *E. coli* O157:H7), WGS data was already available; WGS data were generated for all the other strains and yielded genome sizes from 5.2 to 5.6 Mbp (see Supplementary Table 4 for detailed WGS data). A core SNP maximum likelihood tree showed that strains clustered by serotype (Figure 3). Among all *E. coli* strains, the number of pairwise core SNP differences ranged from 22 to 1,174 SNPs. All strains were also assessed for potential mutations in *rpoS*, which encodes the alternative sigma factor (RpoS); polymorphism in this gene as well as accumulation of mutations in lab strains which may alter stress response have been previously described (Snyder et al., 2012). Among the 17 strains, RpoS was highly conserved with only two polymorphic amino acid sites detected in FSL R9-5257 (D118N), and FSL R9-5513 (G309D). In addition, a single point mutation in strain FSL R9-3467 lead to a frameshift and premature stop codon. For strain FSL R9-5512, the last eight amino acids in the sequence differ from the consensus sequence, due to a 11 bp deletion (nucleotides 966 to 976) at the 3′ end of *rpoS* (see Supplementary Figure 6 for details).

### Effect of Strain Diversity and Pre-growth Conditions on PAA Sensitivity Across Bacterial Groups

As an initial application, our strain collection was used to characterize the die-off of different bacterial strains after short exposure to a produce industry relevant sanitizer (i.e., PAA). For these experiments, a subset of strains was conveniently selected for each bacterial group, including (i) four *L. monocytogenes* and one *L. innocua* (this species represents a possible surrogate for *L. monocytogenes*); (ii) four *S. enterica* and two potential *S. enterica* surrogates (the avirulent *Salmonella* Typhimurium MHM112 and *E. faecium* ATCC 8459) and (iii) five STEC and one potential surrogate (i.e., *E. coli* FSL R9-4077). Pathogen strains were selected to represent phylogenetically distinct strains (based on the core SNP phylogenies, see Figure 1–3) and preference was given to select strains from produce-associated outbreaks. Each strain was pre-grown under each of the seven different conditions to assess the relative impact of strain diversity and growth condition on PAA survival.

To assess the variance of responses due to strain versus the variance of responses due to pre-growth condition, data for each of the three pathogen groups (i.e., *L. monocytogenes, S. enterica*, STEC) were assessed using a crossed random effects model. This model showed that for each pathogen group, the “condition” variance component was larger than the “strain” variance component and the “strain-condition interaction” variance component (Table 2). A larger variance component for condition means that condition contributes the most to the overall response variance. For *L. monocytogenes*, the variance component for “condition” was 64.2 (95% CI = 21.2, 245.2), while the variance components for “strain” and “strain-condition interaction” were 7.4 (95% CI = 0.0, 72.6) and 1.6 (95% CI = 0.0, 28.0), respectively. For *S. enterica*, the variance component for “condition” was 53.0 (95% CI = 0.0, 122.2), while the variance components for “strain” and “strain-condition interaction” were 15.9 (95% CI = 0.0, 51.0) and 26.5 (95% CI = 0.0, 107.4), respectively. For STEC, the variance component for “condition” was 126.7 (95% CI = 13.6, 274.9); while the variance components for “strain” and “strain-condition interaction” were 0.0 (95% CI = 0.0, 9.0) and 0.0 (95% CI = 0.0, 28.0), respectively. The reported variance components for *L. monocytogenes, S. enterica* and STEC indicate that pre-growth conditions have a larger effect on variation in responses than strain diversity.

**Table 2:** Variance components of crossed random effects model for *L. monocytogenes, S. enterica*, and STEC.

Model Variable	Variance Component	95% Confidence Interval
***L. monocytogenes***		
Condition	64.2	21.2, 245.2
Strain	7.4	0.0, 72.6
Strain-Condition Interaction	1.6	0.0, 28.0
***Salmonella***		
Condition	53.0	0.0, 122.2
Strain	15.9	0.0, 51.0
Strain-Condition Interaction	26.5	0.0, 107.4
***E. coli***		
Condition	126.7	13.6, 274.9
Strain	0.0	0.0, 9.0
Strain-Condition Interaction	0.0	0.0, 28.0


### Survival of PAA Exposure by Different *Listeria* Strains Pre-grown Under Different Conditions

As four *L. monocytogenes* strains, each pre-grown under seven different conditions, were evaluated for survival of exposure to 60 ppm PAA for 45 s, die-off data for a total of 28 “strain-condition” combinations were created. Among these “strain-condition” combinations, die-off ranged from a low of 0.5 log (FSL J1-0031, growth under high salt) to a high of 6.4 log (FSL R9-5411, growth in defined minimal medium). The mean die-off rates for different conditions ranged from a low of 1.0 log (for pre-growth under salt stress) to a high of 5.6 log (for pre-growth in minimal media). The range of die-off values observed with different strains pre-grown under a single condition ranged from 0.5 to 1.8 log (1.3 log range) for pre-growth under salt stress to 2.5 to 4.6 log (2.1 log range) for pre-growth at 21°C.

The mean die-off rates for different strains ranged from a low of 2.1 log (for FSL J1-031, lineage III, 4a) to a high of 3.3 log (FSL R9-0506, lineage II, 1/2a). The range of die-off values observed for a given strain pre-grown under different conditions ranged from 0.5 to 4.5 log (4.0 log range) for strain FSL J1-031 to 1.0 to 6.4 log (5.4 log range) for strain FSL R9-5411. Consistent with the model data detailed in the previous section, the ranges of responses observed within a strain pre-grown under different conditions were considerable larger than the ranges of responses observed within a given condition. While this observation could be due to the fact that more conditions than strains were evaluated, the model results detailed above are not affected by the difference in number of strains (*n* = 4) and conditions assessed (*n* = 7).

**FIGURE 4:**
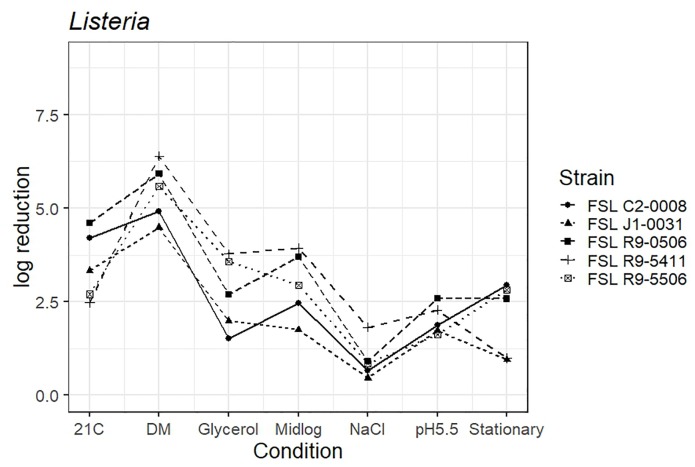
Average log reduction of four *Listeria* strains and one surrogate (*L. innocua*, FSL C2-0008) when pre-grown under different conditions prior to exposure to 60 ppm PAA for 45 s. Pre-growth conditions are shown on the *X*-axis and include pre-growth (i) at 21°C (21C), (ii) in defined minimal medium (DM), (iii) under reduced water activity (Glycerol), (iv) to mid-log phase (Midlog), (v) in 4.5% additional NaCl (NaCl), (vi) at pH 5.5 (pH5.5) and (vii) to stationary phase (Stationary). When calculating log reduction, values with count zero post-sanitizer treatment were substituted with the detection limit (e.g., 100 CFU/mL). Data represent averages from three trials; the standard deviation of the three trials and number of trials with values below detection limit after PAA exposure are listed in Supplementary Table 6.

The average response for the *L. innocua* strain (FSL C2-0008) lies within the range of response of the other strains for five of the conditions (i.e., pre-growth in defined minimal medium, at 21°C, at pH 5.5, to mid-log phase and high salt). When pre-grown to stationary phase *L. innocua* showed a 2.9 log reduction as compared to the average log reduction for the four *L. monocytogenes* strains, which ranged from 0.9 to 2.8 log. When pre-grown in reduced water activity, *L. innocua* showed numerically higher tolerance (1.5 log reduction) as compared to the range observed among the four *L. monocytogenes strains* (log reduction ranged from 2.0 to 3.8 log) (Figure 4; see Supplementary Table 6 for detailed log reduction data).

### Survival of PAA Exposure by Different *S. enterica* Strains Pre-grown Under Different Conditions

As four *S. enterica* strains, each pre-grown under seven different conditions, were evaluated for survival of exposure to 40 ppm PAA for 45 s, die-off data for a total of 28 “strain-condition” combinations were created. Among these “strain-condition” combinations, die-off ranged from a low of 2.6 log (FSL R9-5344, growth under salt stress) to a high of 7.1 log (FSL R9-5272, growth in minimal medium). The mean die-off rates for different conditions ranged from a low of 3.1 log (for pre-growth under salt stress) to a high of 6.0 log (for pre-growth to mid-log phase). The range of die-off values observed with different strains pre-grown under a single condition ranged from 4.9 to 6.4 log (1.5 log range) for pre-growth at low pH to 4.8 to 7.1 log (2.3 log range) for pre-growth in minimal media.

The mean die-off rates for different strains ranged from a low of 4.6 log (for FSL R9-5502, *Salmonella* Poona) to a high of 6.2 log (FSL R9-5272, *Salmonella* Enteritidis). The range of die-off values observed for a given strain pre-grown under different conditions ranged from 4.3 to 7.1 log (2.8 log range) for strain FSL R9-5272 to 2.6 to 6.2 log (3.6 log range) for strain FSL R9-5344. Consistent with the model data detailed in the previous section, the ranges of responses observed within a strain pre-grown under different conditions are considerable larger than the ranges of responses observed within a given condition.

**FIGURE 5:**
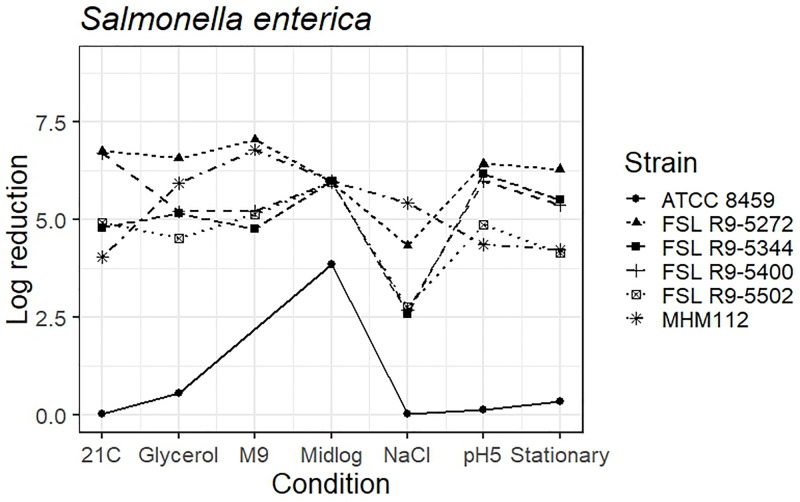
Average log reduction of four *S. enterica* strains and two surrogate strains (avirulent *Salmonella*, MHM112 and *E. faecium*, ATCC8459) when pre-grown under different conditions prior to exposure to 40 ppm PAA for 45 s. Pre-growth conditions are shown on the *X*-axis and include pre-growth (i) at 21°C (21C), (ii) under reduced water activity (Glycerol), (iii) in minimal medium (M9), (iv) to mid-log phase (Midlog), (v) in 4.5% additional NaCl (NaCl), (vi) at pH 5.0 (pH5) and (vii) to stationary phase (Stationary). When calculating log reduction, values with count zero post-sanitizer treatment were substituted with the detection limit (e.g., 100 CFU/mL). Data represent averages from three trials; the standard deviation of the three trials and number of trials with values below detection limit after PAA exposure are listed in Supplementary Table 6.

The average response for the avirulent *S. enterica* strain MHM112 lies within the range of response of the other strains for four of the conditions (i.e., pre-growth in reduced water activity, M9 minimal medium, to mid-log phase and stationary phase). When pre-grown under salt stress, at 21°C and pH 5.0, the avirulent *S. enterica* showed a numerically larger reduction after PAA exposure (5.4 log for salt stress, 4.0 log for 21°C and 4.4 log for pH 5.0) as compared to the average log reduction for the four *S. enterica* strains, which ranged from 2.6 to 4.3 log for salt stress, 4.8 to 6.8 log for 21°C and 4.9 to 6.4 for pH 5.0) (Figure 5 and see Supplementary Table 6 for detailed log reduction data). The average log reduction of surrogate organism *E. faecium* (ATCC 8459) for *S. enterica* was numerically lower across all conditions ranging from 0.0 to 0.08 log when the lowest log reduction for all other *S. enterica* ranged from 2.6 to 4.6 log, not including the 3.9 log reduction for *E. faecium* cells grown to mid-log phase which was also numerically lower than compared to 6.0 log reduction for all other *S. enterica* strains (Figure 5 and Supplementary Table 6). Log reduction data for *E. faecium* in minimal medium was not available due to the lack of growth in the medium.

### Survival of PAA Exposure by Different *E. coli* Strains Pre-grown Under Different Conditions

As five STEC strains, each pre-grown under seven different conditions, were evaluated for survival of exposure to 40 ppm PAA for 45 s, die-off data for a total of 35 “strain-condition” combinations were created. Among these “strain-condition” combinations, die-off ranged from a low of 1.5 log (FSL R9-5271, growth under salt stress) to a high of 6.5 log (FSL R9-5516, FSL R9-5271, growth in minimal medium). The mean die-off rates for different conditions ranged from a low of 2.9 log (for pre-growth under salt stress and at 21°C) to a high of 6.5 log (for pre-growth in minimal media). The range of die-off values observed with different strains pre-grown under a single condition ranged from 5.3 to 6.1 log (0.8 log range) for pre-growth to mid-log phase to 1.5 to 4.2 log (2.7 log range) for pre-growth under salt stress.

The mean die-off rates for different strains ranged from a low of 4.2 log (for FSL R9-5516, O145) to a high of 4.6 log (FSL R9-5517, O103). The range of die-off values observed for a given strain pre-grown under different conditions ranged from 2.9 to 6.1 log (3.2 log range) for strain FSL R9-5258 to 1.5 to 6.5 log (5.0 log range) for strain FSL R9-5271. Consistent with the model data detailed in the previous section, the ranges of responses observed within a strain pre-grown under different conditions are considerable larger than the ranges of responses observed within a given condition.

**FIGURE 6:**
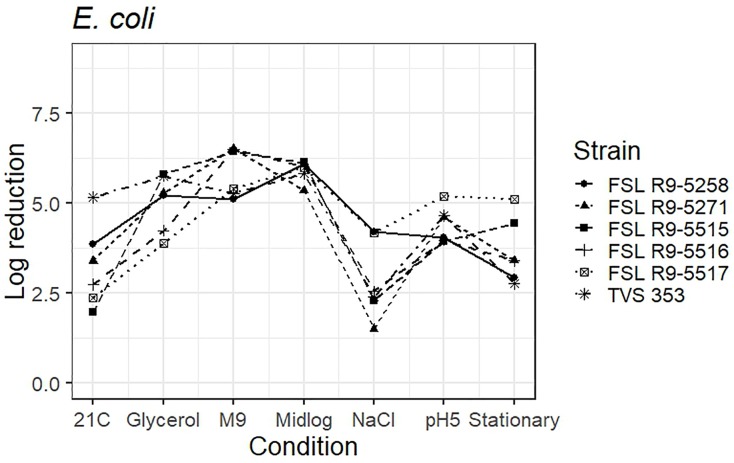
Average log reduction of five STEC strains and one surrogate (*E. coli* strain, TVS 353) when pre-grown under different conditions prior to exposure to 40 ppm PAA for 45 s. Pre-growth conditions are shown on the *X*-axis and include pre-growth (i) at 21°C (21C), (ii) under reduced water activity (Glycerol), (iii) in minimal medium (M9), (iv) to mid-log phase (Midlog), (v) in 4.5% additional NaCl (NaCl), (vi) at pH 5.0 (pH5) and (vii) to stationary phase (Stationary). When calculating log reduction, values with count zero post-sanitizer treatment were substituted with the detection limit (e.g., 100 CFU/mL). Data represent averages from three trials; the standard deviation of the three trials and number of trials with values below detection limit after PAA exposure are listed in Supplementary Table 6.

The average response for the STEC surrogate *E. coli* TVS 353 lies within the range of response of the other strains for six conditions (i.e., pre-grown in reduced water activity, high salt, M9 minimal medium, pH 5.0, to mid-log phase and stationary phase). When pre-grown at 21°C, *E. coli* TVS 353 showed a 5.2 log reduction as compared to the average log reduction for the five STEC strains, which ranged from 2.0 to 3.9 log (Figure 6 and see Supplementary Table 6 for detailed log reduction data).

### Prediction Model Estimates for the Likely Ranges of Reduction After PAA Exposure for Different Combinations of Strains and Pre-growth Conditions

**FIGURE 7:**
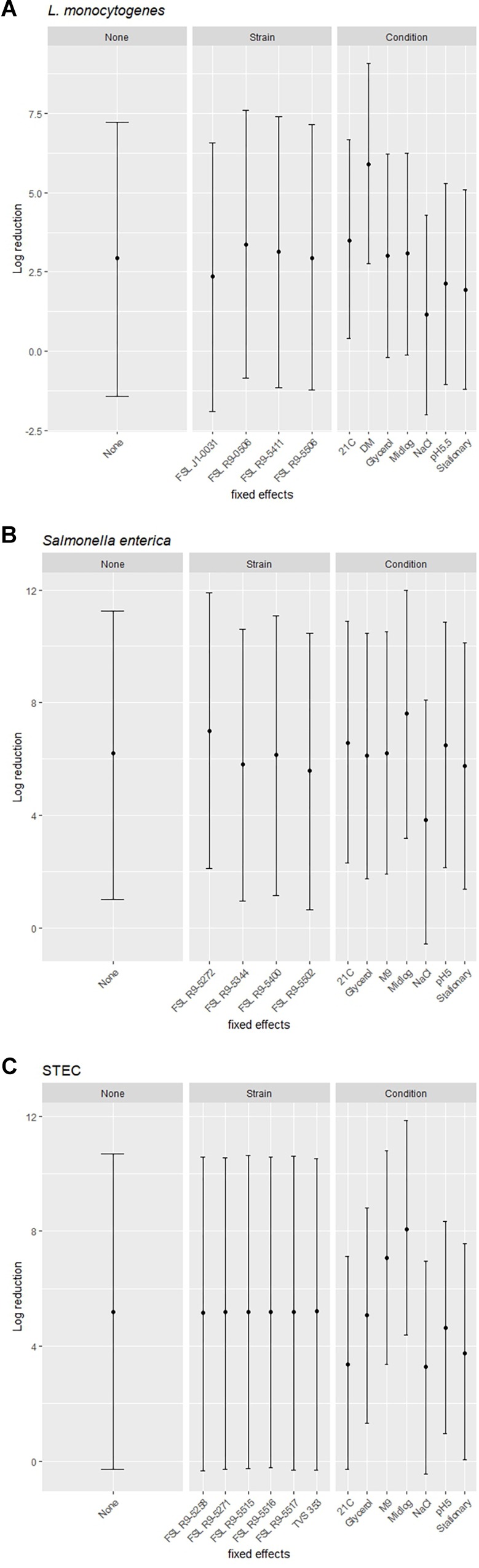
Estimated ranges of log reduction after PAA exposure for different combinations of strains and pre-growth conditions. 95% prediction intervals for log reduction were estimated based on a crossed-random effects model where no effect was fixed (left panel, labeled “None”), the effect of strain was fixed, and conditions were random effects (middle panel, labeled “Strain”), and the effect of condition was fixed and strains were random effects (right panel, labeled “Condition”). The error bars of the 95% prediction intervals show the range of responses in log reduction (*y*-axis) for a given effect after exposure to PAA of **(A)** *L. monocytogenes*, **(B)** *S. enterica*, and **(C)** STEC.

A modeling-based approach was used to assess the range of bacterial reductions expected when different combinations of strains and growth conditions were tested for PAA survival. Specifically, the 95% prediction intervals (PIs) for log reduction were estimated with a cross-random effects model where either (i) no effect was fixed (random combinations of strain and growth condition), (ii) the effect of a single strain was fixed (single strain with random selection of growth condition) or (iii) the effect of a single pre-growth condition was fixed (single condition with random selection of strains). For example, for *L. monocytogenes* when all strains and conditions were chosen at random and none of the effects were fixed, the PI covered a 8.7 log range. By comparison, the experimentally observed responses for *L. monocytogenes* covered a 5.9 log range (from a 0.5 log reduction observed for FSL J1-031 pre-grown under high salt to a 6.4 log-reduction observed for FSL R9-5411 pre-grown in minimal medium). The larger range for the model-based approach is due to the fact that the possible log reduction parameters cover a larger range than the observed log reductions (which represents the arithmetic mean of 3 replicates). The *L. monocytogenes* PIs for single strains pre-grown under different conditions ranged from 8.4 to 8.6 log (depending on strain), while PIs for single pre-growth conditions (each with multiple strains) ranged from a 6.3 to a 6.4 log reduction (depending on growth condition) (Figure 7A). For *S. enterica*, when all strains and conditions were chosen at random and none of the effects were fixed, the PI covered a 9.7 log range, for single strains pre-grown under different conditions PIs ranged from 9.7 to 9.9 log, while PIs for single pre-growth conditions ranged from 8.6 to 8.8 log (Figure 7B). For STEC, when all strains and conditions were chosen at random and none of the effects were fixed, the PI covered a 11.3 log range, for single strains pre-grown under different conditions PIs ranged from 11.1 to 11.3 log, while PIs for single pre-growth conditions ranged from 7.4 to 7.7 log (Figure 7C). Overall, the model predictions further support that assessment of a single strain pre-grown under different conditions captures a larger range of responses as compared to assessment of multiple strains pre-grown under a single condition.

## Discussion

With increasing requirements for science-based food safety strategies, industry needs scientifically sound approaches for validation studies, including selection of pathogen or surrogate strains used and the use of specific protocols for bacterial growth prior to validation studies. There is a particular need for pathogen and surrogate strain collections, as well as growth protocols for the produce industry as validation of intervention and control strategies is particularly important for this industry, which often lacks well established and validated pathogen control steps. Therefore, we assembled a produce-relevant bacterial strain collection that includes key pathogens as well as possible surrogate organisms, followed by WGS to characterize and validate the strains in this collection. In a proof of concept experiment, a subset of strains was further assessed, after pre-growth under different conditions, for survival of PAA exposure. These experiments provided clear evidence that growth conditions prior to challenge studies have a larger effect on phenotypic outcomes than strain diversity, suggesting that the use of a cocktail that contains strains pre-grown under different conditions may provide a valuable alternative to currently more typically used multi-strain cocktails pre-grown under a single condition.

### Assembly of a Unique Produce-Relevant Strain Collection Will Facilitate Challenge and Validation Studies

The produce-relevant strain collection assembled includes 11 *L. monocytogenes*, 23 *S. enterica*, and 13 STEC, as well as 8 possible surrogate organisms. Selection of appropriate surrogate organisms can be controversial since it is necessary to determine whether the chosen organism emulates the target for each different use condition. While additional surrogates may hence need to be added, the current collection should represent an appropriate starting point. For example, *L. innocua* has been well established as a surrogate for *L. monocytogenes* including through studies where representatives of these two species showed similar (i) growth on baby spinach leaves (Omac et al., 2015), (ii) survival rates in soil (Girardin et al., 2005), and (iii) D and *z*-values for heat treatment at various temperatures (Friedly et al., 2008). While microbial strain collections for a variety of pathogens have been established [e.g., ILSI NA *L. monocytogenes* Strain Collection (Fugett et al., 2006); standard reference collection of STEC strains (Manning, 2011)], the collection here represents the first strain collection tailored to produce safety research. Broad use of this standard strain collection will also allow for better comparison of data across different studies. This will be facilitated by the fact that some of the strains in this collection have already been used, including studies of *S. enterica* survival on tomatoes (López-Velasco et al., 2012, 2013), antimicrobial treatments of broccoli and radish seeds (Landry et al., 2015), penetration of *S. enterica* into fruit (Danyluk et al., 2010), *E. coli* survival on lettuce (Bezanson et al., 2012; Weller et al., 2017b), and persistence on tomatoes, cantaloupe and spinach (de Moraes et al., 2016). In addition, initial characterization of selected surrogates in this collection for PAA sensitivity already provides some valuable information. For example, while a comparison of the avirulent *Salmonella* surrogate strain MHM112 with the other *Salmonella for* strains suggests the suitability of this strain as a surrogate organism for PAA treatment, use of *E. faecium* in challenge studies, at least with PAA, would typically underestimate the efficacy of PAA for *Salmonella* as this surrogate strain showed considerably higher PAA resistance as compared to *Salmonella*.

Acquisition of pathogen strains, particularly from international sources, is however, not trivial and hence this collection is biased towards strains from the United States, even though some strains from international sources are included, such as an *E. coli* O104:H4 (FSL R9-5256) from the sprout-associated outbreak in Germany 2011 (Ahmed et al., 2012). Lack of a broad geographical representation could be a particular challenge for *S. enterica*, where it has been established that some serotypes show strong geographical associations; for example, serotype Stanley is predominantly found in Thailand (Hendriksen et al., 2009) and was responsible for an outbreak linked to alfalfa sprouts (Werner et al., 2007). One could also consider including additional *L. monocytogenes* strains that represent the most prevalent clonal complexes (CC) worldwide for each lineage e.g., CC1, CC2 and CC3 for lineage I, as well as CC7 and CC9 for lineage II (Chenal-Francisque et al., 2011). Moreover, the collection could be extended to include spoilage organisms such as *Pseudomonas* strains to assess the effectiveness of sanitizer against (i) spoilage organisms or (ii) foodborne pathogens present in mixed biofilms that include *Pseudomonas*. While the strain collection described here provides a valuable starting point, it is anticipated that in the future other strains (e.g., from new outbreaks, international sources) will be added and that individual users may supplement the collection with specific strains of interest (e.g., strains previously linked to a facility or recalls of interest).

### WGS Is Essential for Unambiguous Identification of Strains

All strains included in the collection were characterized by WGS using KRAKEN and kSNP to confirm strain identity and to classify strains based on their genetic relationships, respectively. WGS data will also allow for future unambiguous identification of strains and strain verification, which is crucial when strains are distributed to other researchers with the associated risk of cross-contamination or mislabeling. This challenge was confirmed here as two isolates received did not match the expected serotype and needed to be re-acquired from a third party. Similarly, previous studies (Goto, 1992; Stacey, 2000; Madisch and Heim, 2007) have shown for bacterial isolates and viruses that mislabeling can occur at culture collections for type strains. Availability of a strain collection with associated publicly available WGS data will allow users to validate every strain upon receipt and/or over time, as well as after completion of experiments. For example, after PAA exposure surviving strains could have been characterized by WGS to identify if the sanitizer is selecting for strains with spontaneous mutations that confer an advantage. WGS data could also be used to develop strain specific primers, which would be valuable if confirmation of recovered challenge strains is needed (e.g., in field studies) (Tomás-Callejas et al., 2011; Naganandhini et al., 2015). Not unexpected, some genome sequence data represented a considerable number of contigs (>100), including all *E. coli* strains, which is consistent with previous observations that assembly of *E. coli* genomes from short read sequencing data is challenging, presumably due to the presence of a larger number of repeat regions (Higgins et al., 1982; Gilson et al., 1984; Brem, 2000; Williams et al., 2013). Future users of this collection may hence select to re-sequence some of the strains in this collection with long-read sequencing methods, e.g., PACBIO.

Whole genome sequencing data for strains included in a collection also provides an opportunity to screen strains for unusual mutations that may lead to phenotypes that are not representative of a specific species or serotype. Initial WGS-based screen and quality checks could include (i) identification of genes with premature stop codons, (ii) identification of non-synonymous changes in functional regions of key genes (e.g., stress response genes), and (iii) partial or full deletions of key genes. Identification of mutations that affect key phenotypes is particularly important if strains will be used for challenge studies, where mutations that affect stress response mechanisms may lead to overestimation of the effectiveness of an intervention. Bioinformatic evaluation of strains for mutations in key stress response (and virulence genes) is also important as many strains may have been passaged for a number of generations in rich media and hence are more likely to have undergone adaptation to a laboratory environment (Eydallin et al., 2014; Liu et al., 2017). Importantly, we identified premature stops codons in *rpoS*, which encodes a global stress regulator, in one *Salmonella* and one *E. coli* strain; mutations in this gene have previously been shown to lead to reduced stress resistance (Leenanon and Drake, 2001; Kabir et al., 2004). A previous study of human, spinach, and environmental *E. coli* O157:H7 isolates associated with the 2006 spinach outbreak in the United States, also reported that three human isolates as well as two spinach isolates tested carried *rpoS* mutations and showed considerably reduced acid tolerance as compared to environmental and spinach isolates without *rpoS* mutations (Parker et al., 2012).

Strains can also be modified to ease the recovery of a specific strain from a mixed-sample e.g., selection for antibiotic resistance or incorporation of antibiotic resistance genes into the chromosome. Three strains in the collection (FSL R9-5251, FSL R9-5220, FSL R9-6567) had been selected for resistance to nalidixic acid or rifampicin; for these antibiotics, a single point mutation is sufficient to confer resistance. However, antibiotic resistance can lead to pleiotropic effects resulting in dramatic changes in the phenotype which has to be considered when drawing conclusions from challenge or validation studies using the antibiotic resistant strain instead of the wildtype. Also, multiple additional mutations may be present in strains selected for antibiotic resistance, including compensatory mutations that occurred subsequently to the mutation that conferred antibiotic resistance (Robinson et al., 2015; Collery et al., 2017). Indeed, additional non-synonymous mutations were identified in two out of three strains. For example, in strain FSL R9-6567, a non-synonymous change was found in *flh*E, which is a periplasmatic protein that regulates flagellar biosynthesis. As deletion of *flh*E in *S. enterica* has previously been shown to cause a proton leak and changes in the outer membrane (Lee et al., 2015), it may be necessary to assess the consequences of this (or other) non-synonymous mutations before including strains in experimental studies.

### Pre-growth Conditions Tested Lead to Larger Range of Phenotypic Response Than Strain Diversity

Currently, challenge and validation studies are typically conducted with multiple strains, which may be used separately or as a mixture of multiple strains (so called “cocktails”) (Scott et al., 2005). This approach allows to account for strain diversity and to assure that control strategies are designed to deliver the appropriate or required protection even with more tolerant strains. Strains are often selected to represent outbreaks or food sources relevant to a given challenge study (e.g., studies on lettuce would use strains from lettuce or lettuce-associated outbreaks) and strains typically are pre-grown under a single condition. In this study, it was shown that pre-growth conditions have a larger effect on the range of phenotypic responses than strain diversity. Inclusion of different pre-growth conditions in challenge and validation studies is particularly important if it is not possible to define the specific physiological status of bacteria in the natural environments and contamination events relevant for a given challenge study, which may often be the case in the produce industry.

However, the importance of including strain diversity in challenge studies cannot be neglected. In this study, the importance of strain diversity was particularly evident for *S. enterica*, where even though the variance component for “condition” was very large, the variance component for “strain-condition interaction” was larger than for the other two pathogen groups, indicating that the interaction between strain diversity and pre-growth conditions had an impact on PAA survival. These findings for *S. enterica* could at least be partially due to the fact that the *S. enterica* strains included in the collection appear to represent larger genomic diversity (as, for example, supported by larger variation in genome size, suggesting a larger accessory genome) than the STEC and *L. monocytogenes* strains. While a number of previous studies have also shown strain variation with regard to bacterial survival of stress conditions e.g., salt or acid stress (Lianou et al., 2006; Hingston et al., 2017), in many cases this is driven by a few strains that showed extremely high sensitivity to a given stress, which often could be tracked to mutations in key genes (e.g., stress response genes) (Parker et al., 2012; Kovacevic et al., 2013). In other cases, strains do clearly differ in their tolerance to food relevant stress conditions, even though the magnitude of variation is often comparatively small and therefore may be of limited practical relevance. For example, among 101 *L. monocytogenes* isolates the minimum inhibitory concentration (MIC) to benzalkonium chloride, a quaternary ammonium compound, ranged from 5 to 13 ppm for isolates containing the resistance genes *qacH* or *bcrABC*, while isolates without these genes showed MICs ≤ 5 ppm (Møretrø et al., 2017). While this suggests that strains with and without these resistance genes should have a low tolerance to quaternary ammonium at the typical use concentrations, which are at least 200 ppm, one could argue that reduced sensitivity to low quaternary ammonium concentrations could still be relevant as pathogens in processing plants may sometimes only be reached by diluted sanitizer.

There are, however, also a few clear examples of foodborne pathogen strains that show considerably enhanced tolerance to specific food-associated stress conditions, such as *S. enterica* and *E. coli* strains that encode a heat resistance islet (LHR), which appears to considerably enhance heat resistance; up to 3 log decreased survival of heat stress was reported in *E. coli* when one of the three genes *yfdX1, yfdX2*, and *hdeD* on LHR1 were deleted (Mercer et al., 2017). Even though the contribution of strain diversity to phenotypic variation are on average smaller than the contributions of growth conditions, inclusion of highly tolerant strains in challenge studies as well as inclusion of strain diversity thus remains important. This leads to the proposal that future challenge studies should consider including multiple strains, but with each pre-grown under a different condition. If conditions that organisms are typically exposed to are known and well defined (e.g., dry inoculation for nuts [Blessington et al., 2013]), these conditions should be used for or included among the pre-growth conditions. Similarly, if a specific sanitizer is repeatedly used in a processing facility, validation studies should assess if pathogens are adapting to low level concentrations of the sanitizer over time. For experiments on sanitizer survival, it may also be appropriate to include pre-growth under exposure to sub-lethal sanitizer levels. Overall, if “typical” pre-growth conditions are unknown or cannot easily be defined, pre-growth under different conditions, focusing on those that have shown to increase stress tolerance, may be appropriate, which is consistent with prior recommendations to grow the inoculum to stationary phase, which typically leads to more stress tolerant bacterial cells (Beuchat et al., 2001).

Interestingly, in this study strains consistently showed highest PAA tolerance when pre-grown under high salt (mean die-off was 1.0 log for *L. monocytogenes*, 3.1 log for *S. enterica* and 2.9 log for STEC) and least tolerance when pre-grown in minimal medium (mean die-off 5.6 log for *L. monocytogenes* and 6.0 log for STEC) or mid-log phase (mean die-off 6.0 log for *S. enterica*). These findings are consistent with a number of studies that have shown cross-protection of bacteria exposed to one stress (e.g., salt stress) to subsequent exposure to another stress (e.g., oxidative stress). For example, *L. monocytogenes* pre-adapted in 6% NaCl became more tolerant when exposed to 50 mM H_2_O_2_ than compared to control cultures (Bergholz et al., 2012). In another study, pre-growth of *L. monocytogenes* under osmotic stress lead to increased heat resistance (Jørgensen et al., 1995), as well as nisin and bile salt tolerance (Begley et al., 2002; Bergholz et al., 2013). Examples of cross-protection, by other stresses, against additional food relevant stress conditions include increased heat resistance after acid adaptation in *S. enterica* and *E. coli* (Leyer and Johnson, 1993; Haberbeck et al., 2017), increased survival of filamentous *S. enterica* in low pH and during desiccation, when pre-grown in reduced water activity (Stackhouse et al., 2012). However, exposure to one stress, does not always provide enhanced tolerance to another stress; for example, it has been shown for *S. enterica* that adaptation to acid stress leads to increased sensitivity to subsequent oxidative stress (Greenacre et al., 2006) due to the downregulation of the transcription factor OxyR, which is crucial in oxidative stress response (Christman et al., 1985). Overall, this supports the importance of pre-growth under multiple conditions rather than focusing on pre-growth in a single condition (e.g., salt), as different pre-growth conditions may enhance tolerance to different given interventions. Our data indicate though that certain pre-growth conditions (e.g., mid-log phase, minimal media) may generally lead to less stress tolerant bacterial cells and hence should potentially be excluded as an appropriate pre-growth condition. This is consistent with data that show reduced stress tolerance of log phase cells during osmotic stress as compared to stationary phase cells (Jenkins et al., 1990). Similarly, reduced stress tolerance to high osmolarity had previously been shown for *L. monocytogenes* that was pre-grown in defined minimal medium as compared to pre-growth in BHI (Maria-Rosario et al., 1995). This is likely due to accumulation of compatible solutes, such as glycine betaine and carnitine, that are present in full media (e.g., BHI); accumulation of these compounds helps stabilize enzymes and proteins, thus ensuring their continuous function in adverse conditions (Mendum and Smith, 2002).

## Conclusion

Overall, our data indicate that conditions used to grow bacterial strains prior to challenge studies have, on average, a larger effect on challenge study outcomes and survival as compared to strain diversity. Strain diversity parameters that may affect stress tolerance and survival of interventions can often be linked to mutations in stress response genes that can be easily identified with appropriate bioinformatic approaches as long as genome sequence data are available, as is the case for all strains included in the collection described here. However, strains that show “hyper-tolerance” to certain stress conditions do exist and their inclusion in challenge sets is important, particularly if challenge studies are intended to identify “worst case scenarios” (i.e., highest resistance to be expected among naturally occurring pathogen strains). Based on the data available to date, we suggest that challenge studies may want to utilize a 5-strain cocktail with (i) each strain confirmed to not have known mutations in key relevant stress response and other genes and where (ii) each strain is pre-grown under a different condition (possibly excluding conditions that are well established to yield hyper-tolerant strains, e.g., log phase); under some circumstances, random number generators could be used to select the 5 conditions to be used from a larger set of possible and valid pre-growth conditions. In addition, where existence of strains that are hyper-tolerant has been established, these should be included when appropriate for the stress condition or intervention evaluated. However, consistent with prior recommendations, if a given challenge study targets a product pathogen combination where pathogens are expected to be in very specific and well defined physiological state (e.g., low water activity in dry foods), pre-adaptation of all strains in cocktail to these conditions would typically still be warranted.

## Data Availability

The datasets and code for analyses for this study can be found in the CPS-strain-collection repository on github (https://github.com/FiaHa/CPS-strain-collection).

## Author Contributions

AH, VG-O, and MW designed the study. AH conducted the experiments. AH, JK, LC, and DK performed the bioinformatical and statistical analyses. AH and MW wrote the manuscript.

## Conflict of Interest Statement

The authors declare that the research was conducted in the absence of any commercial or financial relationships that could be construed as a potential conflict of interest.

## Footnotes

1 https://www.cdc.gov/pulsenet/pdf/PNL32-MiSeq-Nextera-XT.pdf

2 http://www.bioinformatics.babraham.ac.uk/projects/fastqc

3 http://www.denglab.info/SeqSero

4 http://tree.bio.ed.ac.uk/software/figtree/

5 http://www.foodmicrobetracker.com
