# Supplementary material for: Assembly and Characterization of a Pathogen Strain Collection for Produce Safety Applications: Pre-growth Conditions Have a Larger Effect on Peroxyacetic Acid Tolerance Than Strain Diversity
Source: Front Microbiol. 2019 May 31;10:1223. doi: 10.3389/fmicb.2019.01223 (PMC6558390; doi:10.3389/fmicb.2019.01223)
Supplement: Supplementary file 1 [file Table_4.pdf]

Supplemental table 4: WGS data for all sequenced strains

Listeria monocytogenes				
FSL ID	Serotype	Genome size	Coverage	Number of contigs
FSL J1-108	4b	3	121.5	26
FSL R2-503	1/2b	3	81.1	309
FSL J1-107	4d	3	183.8	24
FSL J1-101	1/2a	3	181.2	36
FSL R9-0506	1/2a	2.9	45.6	90
FSL J1-031	4a	2.8	125.3	29
FSL J1-158	4b	2.9	181.6	35
FSL S10-2161	1/2a	2.9	233.7	23
Salmonella enterica				
FSL R9-5400	Saintpaul	4.7	85.4	106
FSL R9-5402	Tennessee	4.8	40.4	107
FSL R9-5494	Typhimurium	4.9	117.9	57
FSL R9-5409	Typhimurium	5	90.5	157
FSL R9-6568	Poona	4.7	102.4	125
FSL R9-6569	Poona – R*	4.7	156.4	67
FSL R9-5272	Enteritidis - PT30	4.8	114.5	89
FSL R9-5273	Javiana	4.6	97.0	92
FSL R9-5251	Newport – R*	4.8	146.2	88
FSL R9-5252	Newport (antimicrobial susceptible)	4.8	255.7	60
FSL R9-5274	Senftenberg 775W	5.3	151.7	197
FSL R9-5496	I 4,[5],12:i:-	4.9	117.8	69
FSL R9-5344	Litchfield	4.6	40.4	351
FSL R9-5502	Poona – N*	4.5	25.6	351
FSL R9-5219	Anatum	4.8	131.8	65
FSL R9-5220	Anatum – N*	4.8	114.5	57
FSL R9-5497	Infantis	4.6	94.9	88
FSL R9-5498	Muenchen	4.7	85.9	72
FSL R9-5499	I 13,23:b:-	4.6	81.8	112
FSL R9-5504	Newport (MDR)	4.9	70.4	282
FSL R9-5406	Montevideo	4.7	40.4	96
FSL R9-5505	Enteriditis	4.9	260.9	198

		Escherichia coli		
FSL R9-5509	O121:H19	5.3	76.2	363
FSL R9-5257	O104:H4	5.3	68.3	270
FSL R9-5258	O104:H4	5.4	94.8	269
FSL R9-5512	O26:H11	5.5	100.4	423
FSL R9-5271	O157:H7	5.3	87.1	338
FSL R9-5513	O157:H7	5.5	74.9	295
FSL R9-5345	O111:H8	5.5	102.2	621
FSL R9-5515	O111:H8	5.2	67.3	333
FSL R9-5517	O103:H2	5.5	68.2	320
FSL R9-5639	O26:H11	5.6	34.0	436
FSL R9-6071	O45:H2	5.2	98.5	650
		Surrogate organisms		
FSL C2-0008	*Listeria innocua*	2.8	198.8	14
FSL R9-4077	*E. coli* O88:H25	5.0	104.1	143
FSL R9-4078	*E. coli* O88:H25	5.0	87.1	137
FSL R9-4079	*E. coli* O88:H25	5.0	84.0	149
