# Supplementary material for: Assembly and Characterization of a Pathogen Strain Collection for Produce Safety Applications: Pre-growth Conditions Have a Larger Effect on Peroxyacetic Acid Tolerance Than Strain Diversity
Source: Front Microbiol. 2019 May 31;10:1223. doi: 10.3389/fmicb.2019.01223 (PMC6558390; doi:10.3389/fmicb.2019.01223)
Supplement: Supplementary file 2 [file Data_Sheet_1.PDF]

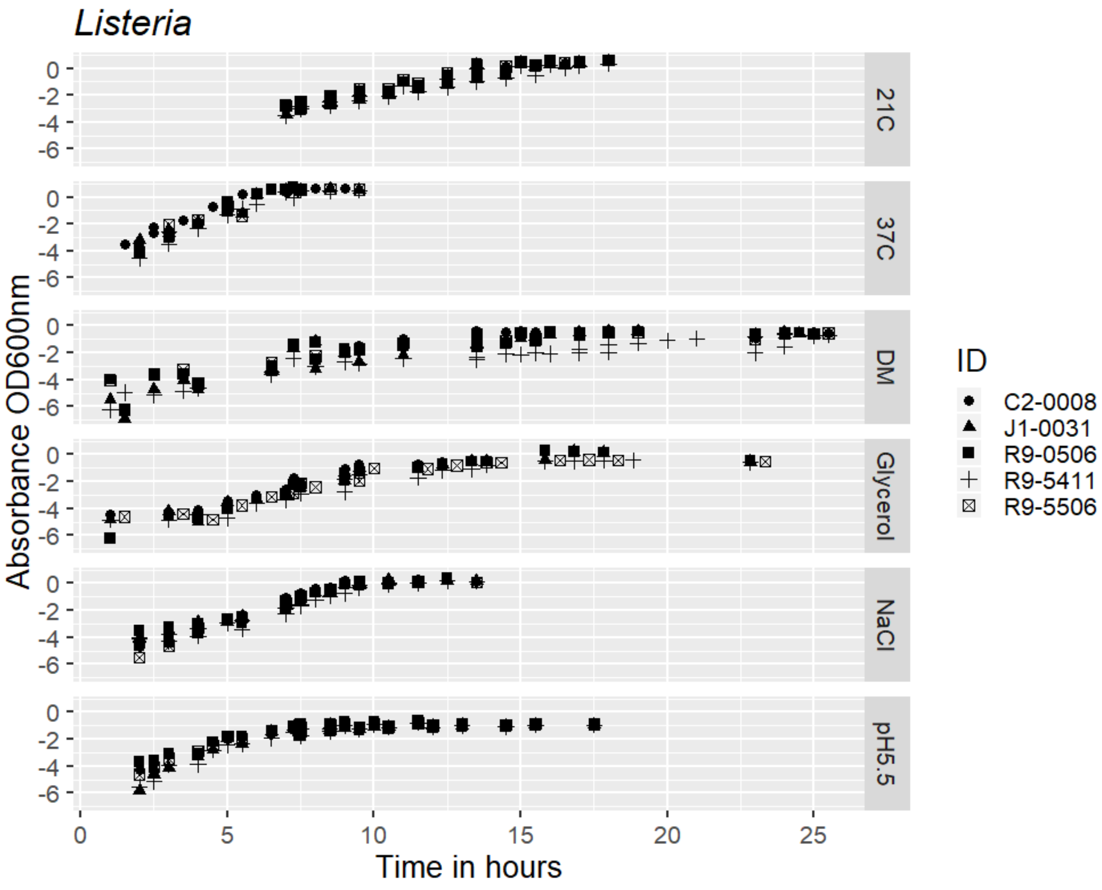

S Figure 1. Growth curves for *Listeria* strains for each pre-growth condition including pre-growth at 21°C (21C), at 37°C (37C), in defined medium (DM), reduced water activity (Glycerol), in 4.5% additional NaCl (NaCl), and pH 5.5 (pH5.5) (rows). Measured absorbance at OD600nm was log transformed (y-axis) and plotted in hour (x-axis).
