# Supplementary material for: Assembly and Characterization of a Pathogen Strain Collection for Produce Safety Applications: Pre-growth Conditions Have a Larger Effect on Peroxyacetic Acid Tolerance Than Strain Diversity
Source: Front Microbiol. 2019 May 31;10:1223. doi: 10.3389/fmicb.2019.01223 (PMC6558390; doi:10.3389/fmicb.2019.01223)
Supplement: Supplementary file 4 [file Data_Sheet_3.PDF]

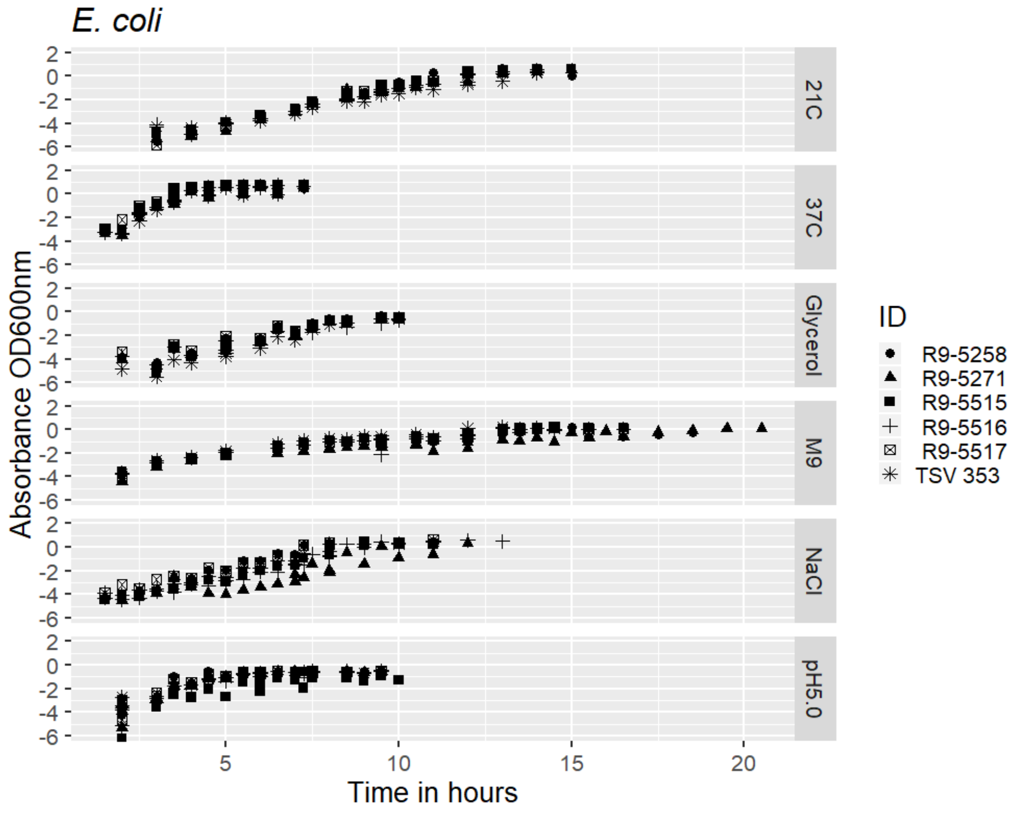

S Figure 3. Growth curves for *E. coli* strains for each pre-growth condition including pre-growth at 21°C (21C), at 37°C (37C), reduced water activity (Glycerol), in minimal medium (M9), in 4.5% additional NaCl (NaCl), and pH 5.0 (pH5.0) (rows). Measured absorbance at OD600nm was log transformed (y-axis) and plotted in hour (x-axis).
