# Supplementary material for: Assembly and Characterization of a Pathogen Strain Collection for Produce Safety Applications: Pre-growth Conditions Have a Larger Effect on Peroxyacetic Acid Tolerance Than Strain Diversity
Source: Front Microbiol. 2019 May 31;10:1223. doi: 10.3389/fmicb.2019.01223 (PMC6558390; doi:10.3389/fmicb.2019.01223)
Supplement: Supplementary file 5 [file Data_Sheet_4.PDF]

S Figure 4: Muscle alignment of sigB amino acid sequences of *Listeria*

```
               1        10        20        30        40        50        60
               |         |         |         |         |         |         |
  FSL C2-0008  MPKVSQPDKEAKEKVYIWIAAYQEDGDEDAQYNLVIHYKNLVESIARKYSQGKSFHEDLV
   FSL J1-158  MPKVSQPDKEAKEKVYIWIAAYQENGDEDAQYNLVVHYKNLVESIARKYSQGKSFHEDLV
   FSL J1-031  MPKVSQPDKEAKEKVYIWIAAYQENGDQDAQYNLVVHYKNLVESIARKYSQGKSFHEDLV
   FSL J1-107  MPKVSQPDKEAKEKVYIWIAAYQENGDQDAQYNLVVHYKNLVESIARKYSQGKSFHEDLV
   FSL J1-108  MPKVSQPDKEAKEKVYIWIAAYQENGDQDAQYNLVVHYKNLVESIARKYSQGKSFHEDLV
   FSL R2-503  MPKVSQPDKEAKEKVYIWIAAYQENGDQDAQYNLVVHYKNLVESIARKYSQGKSFHEDLV
  FSL R9-5411  MPKVSQPDKEAKEKVYIWIAAYQENGDQDAQYNLVVHYKNLVESIARKYSQGKSFHEDLV
  FSL R9-5506  MPKVSQPDKEAKEKVYIWIAAYQENGDQDAQYNLVVHYKNLVESIARKYSQGKSFHEDLV
  FSL R9-5507  MPKVSQPDKEAKEKVYIWIAAYQENGDQDAQYNLVVHYKNLVESIARKYSQGKSFHEDLV
   FSL J1-101  MPKVSQPDKEAKEKVYIWIAAYQENGDQDAQYNLVVHYKNLVESIARKYSQGKSFHEDLV
  FSL R9-0506  MPKVSQPDKEAKEKVYIWIAAYQENGDQDAQYNLVVHYKNLVESIARKYSQGKSFHEDLV
 FSL S10-2161  MPKVSQPDKEAKEKVYIWIAAYQENGDQDAQYNLVVHYKNLVESIARKYSQGKSFHEDLV

  FSL C2-0008  QVGNIGLLGAIRRYDATFGKSFEAFAVPTIVGEIKRFLRDKTWSVHVPRRIKELGPKIKN
   FSL J1-158  QVGNIGLLGAIRRYDATFGKSFEAFAVPTIVGEIKRFLRDKTWSVHVPRRIKELGPKIKN
   FSL J1-031  QVGNIGLLGAIRRYDATFGKSFEAFAVPTIVGEIKRFLRDKTWSVHVPRRIKELGPKIKN
   FSL J1-107  QVGNIGLLGAIRRYDATFGKSFEAFAVPTIVGEIKRFLRDKTWSVHVPRRIKELGPKIKN
   FSL J1-108  QVGNIGLLGAIRRYDATFGKSFEAFAVPTIVGEIKRFLRDKTWSVHVPRRIKELGPKIKN
   FSL R2-503  QVGNIGLLGAIRRYDATFGKSFEAFAVPTIVGEIKRFLRDKTWSVHVPRRIKELGPKIKN
  FSL R9-5411  QVGNIGLLGAIRRYDATFGKSFEAFAVPTIVGEIKRFLRDKTWSVHVPRRIKELGPKIKN
  FSL R9-5506  QVGNIGLLGAIRRYDATFGKSFEAFAVPTIVGEIKRFLRDKTWSVHVPRRIKELGPKIKN
  FSL R9-5507  QVGNIGLLGAIRRYDATFGKSFEAFAVPTIVGEIKRFLRDKTWSVHVPRRIKELGPKIKN
   FSL J1-101  QVGNIGLLGAIRRYDATFGKSFEAFAVPTIVGEIKRFLRDKTWSVHVPRRIKELGPKIKN
  FSL R9-0506  QVGNIGLLGAIRRYDATFGKSFEAFAVPTIVGEIKRFLRDKTWSVHVPRRIKELGPKIKN
 FSL S10-2161  QVGNIGLLGAIRRYDATFGKSFEAFAVPTIVGEIKRFLRDKTWSVHVPRRIKELGPKIKN

  FSL C2-0008  AVEELTRELQSSPQISDIADFIGVTEEEVLEAMEMGKSYQALSVDHSIEADSDGSTITLL
   FSL J1-158  AVEELTRELQSSPQISDIADFIGVTEEEVLEAMEMGKSYQALSVDHSIEADSDGSTITLL
   FSL J1-031  AVEELTRELQSSPQISDIADFIGVTEEEVLEAMEMGKSYQALSVDHSIEADSDGSTITLL
   FSL J1-107  AVEELTRELQSSPQISDIADFIGVTEEEVLEAMEMGKSYQALSVDHSIEADSDGSTITLL
   FSL J1-108  AVEELTRELQSSPQISDIADFIGVTEEEVLEAMEMGKSYQALSVDHSIEADSDGSTITLL
   FSL R2-503  AVEELTRELQSSPQISDIADFIGVTEEEVLEAMEMGKSYQALSVDHSIEADSDGSTITLL
  FSL R9-5411  AVEELTRELQSSPQISDIADFIGVTEEEVLEAMEMGKSYQALSVDHSIEADSDGSTITLL
  FSL R9-5506  AVEELTRELQSSPQISDIADFIGVTEEEVLEAMEMGKSYQALSVDHSIEADSDGSTITLL
  FSL R9-5507  AVEELTRELQSSPQISDIADFIGVTEEEVLEAMEMGKSYQALSVDHSIEADSDGSTITLL
   FSL J1-101  AVEELTRELQSSPQISDIADFIGVTEEEVLEAMEMGKSYQALSVDHSIEADSDGSTITLL
  FSL R9-0506  AVEELTRELQSSPQISDIADFIGVTEEEVLEAMEMGKSYQALSVDHSIEADSDGSTITLL
 FSL S10-2161  AVEELTRELQSSPQISDIADFIGVTEEEVLEAMEMGKSYQALSVDHSIEADSDGSTITLL

  FSL C2-0008  DVVGGTDDGFERVNQRMLLEKVLPVLDEREQKILQYTFIENRSQKETGELLDISQMHVSR
   FSL J1-158  DVVGGTDDGFERVNQRMLLEKVLPVLDEREQKILQFTFIENRSQKETGELLDISQMHVSR
   FSL J1-031  DVVGGTDDGFERVNQRMLLEKVLPVLDEREQKILQFTFIENRSQKETGELLDISQMHVSR
   FSL J1-107  DVVGGTDDGFERVNQRMLLEKVLPVLDEREQKILQFTFIENRSQKETGELLDISQMHVSR
   FSL J1-108  DVVGGTDDGFERVNQRMLLEKVLPVLDEREQKILQFTFIENRSQKETGELLDISQMHVSR
   FSL R2-503  DVVGGTDDGFERVNQRMLLEKVLPVLDEREQKILQFTFIENRSQKETGELLDISQMHVSR
  FSL R9-5411  DVVGGTDDGFERVNQRMLLEKVLPVLDEREQKILQFTFIENRSQKETGELLDISQMHVSR
  FSL R9-5506  DVVGGTDDGFERVNQRMLLEKVLPVLDEREQKILQFTFIENRSQKETGELLDISQMHVSR
  FSL R9-5507  DVVGGTDDGFERVNQRMLLEKVLPVLDEREQKILQFTFIENRSQKETGELLDISQMHVSR
   FSL J1-101  DVVGGTDDGFERVNQRMLLEKVLPVLDEREQKILQYTFIENRSQKETGELLDISQMHVSR
  FSL R9-0506  DVVGGTDDGFERVNQRMLLEKVLPVLDEREQKILQYTFIENRSQKETGELLDISQMHVSR
 FSL S10-2161  DVVGGTDDGFERVNQRMLLEKVLPVLDEREQKILQYTFIENRSQKETGELLDISQMHVSR
```

FSL C2-0008	IQRQAIKKLREALQNEEVE
FSL J1-158	IQRQAIKKLREALQNEEVE
FSL J1-031	IQRQAIKKLREALQNEEVE
FSL J1-107	IQRQAIKKLREALQNEEVE
FSL J1-108	IQRQAIKKLREALQNEEVE
FSL R2-503	IQRQAIKKLREALQNEEVE
FSL R9-5411	IQRQAIKKLREALQNEEVE
FSL R9-5506	IQRQAIKKLREALQNEEVE
FSL R9-5507	IQRQAIKKLREALQNEEVE
FSL J1-101	IQRQAIKKLREALQNEEVE
FSL R9-0506	IQRQAIKKLREALQNEEVE
FSL S10-2161	IQRQAIKKLREALQNEEVE
