# Supplementary material for: Assembly and Characterization of a Pathogen Strain Collection for Produce Safety Applications: Pre-growth Conditions Have a Larger Effect on Peroxyacetic Acid Tolerance Than Strain Diversity
Source: Front Microbiol. 2019 May 31;10:1223. doi: 10.3389/fmicb.2019.01223 (PMC6558390; doi:10.3389/fmicb.2019.01223)
Supplement: Supplementary file 6 [file Data_Sheet_5.PDF]

S Figure 5: Muscle alignment of rpoS amino acid sequences of *Salmonella*

```
               1       10        20        30        40        50        60
               |       |         |         |         |         |         |
FSL R95505   MSQNTLKVHDLNEDAEFDENGVEAFDEKALSEEEPSDNDLAEEELLSQGATQRVLDATQL
FSL R95220   MSQNTLKVHDLNEDAEFDENGVEAFDEKALSEEEPSDNDLAEEELLSQGATQRVLDATQL
FSL R95250   MSQNTLKVHDLNEDAEFDENGVEAFDEKALSEEEPSDNDLAEEELLSQGATQRVLDATQL
FSL R95251   MSQNTLKVHDLNEDAEFDENGVEAFDEKALSEEEPSDNDLAEEELLSQGATQRVLDATQL
FSL R95272   MSQNTLKVHDLNEDAEFDENGVEAFDEKALSEEEPSDNDLAEEELLSQGATQRVLDATQL
FSL R95273   MSQNTLKVHDLNEDAEFDENGVEAFDEKALSEEEPSDNDLAEEELLSQGATQRVLDATQL
FSL R95274   MSQNTLKVHDLNEDAEFDENGVEAFDEKALSEEEPSDNDLAEEELLSQGATQRVLDATQL
FSL R95344   MSQNTLKVHDLNEDAEFDENGVEAFDEKALSEEEPSDNDLAEEELLSQGATQRVLDATQL
FSL R95400   MSQNTLKVHDLNEDAEFDENGVEAFDEKALSEEEPSDNDLAEEELLSQGATQRVLDATQL
FSL R95402   MSQNTLKVHDLNEDAEFDENGVEAFDEKALSEEEPSDNDLAEEELLSQGATQRVLDATQL
FSL R95406   MSQNTLKVHDLNEDAEFDENGVEAFDEKALSEEEPSDNDLAEEELLSQGATQRVLDATQL
FSL R95409   MSQNTLKVHDLNEDAEFDENGVEAFDEKALSEEEPSDNDLAEEELLSQGATQRVLDATQL
FSL R95495   MSQNTLKVHDLNEDAEFDENGVEAFDEKALSEEEPSDNDLAEEELLSQGATQRVLDATQL
FSL R95496   MSQNTLKVHDLNEDAEFDENGVEAFDEKALSEEEPSDNDLAEEELLSQGATQRVLDATQL
FSL R95497   MSQNTLKVHDLNEDAEFDENGVEAFDEKALSEEEPSDNDLAEEELLSQGATQRVLDATQL
FSL R95498   MSQNTLKVHDLNEDAEFDENGVEAFDEKALSEEEPSDNDLAEEELLSQGATQRVLDATQL
FSL R95504   MSQNTLKVHDLNEDAEFDENGVEAFDEKALSEEEPSDNDLAEEELLSQGATQRVLDATQL
FSL R96567   MSQNTLKVHDLNEDAEFDENGVEAFDEKALSEEEPSDNDLAEEELLSQGATQRVLDATQL
FSL R95252   MSQNTLKVHDLNEDAEFDENGVEAFDEKALSEEEPSDNDLAEEELLSQGATQRVLDATQL
FSL R95494   MSQNTLKVHDLNEDAEFDENGVEAFDEKALSEEEPSDNDLAEEELLSQGATQRVLDATQL
FSL R95499   MSQNTLKVHDLNEDAEFDENGVEAFDEKALSEEEPSDNDLAEEELLSQGATQRVLDATQL
FSL R95502   MSQNTLKVHDLNEDAEFDENGVEAFDEKALSEEEPSDNDLAEEELLSQGATQRVLDATQL

FSL R95505   YLGEIGYSPLLTAEEEVYFARRALRGDVASRRRMIESNLRLVVKIARRYGNRGLALLDLI
FSL R95220   YLGEIGYSPLLTAEEEVYFARRALRGDVASRRRMIESNLRLVVKIARRYGNRGLALLDLI
FSL R95250   YLGEIGYSPLLTAEEEVYFARRALRGDVASRRRMIESNLRLVVKIARRYGNRGLALLDLI
FSL R95251   YLGEIGYSPLLTAEEEVYFARRALRGDVASRRRMIESNLRLVVKIARRYGNRGLALLDLI
FSL R95272   YLGEIGYSPLLTAEEEVYFARRALRGDVASRRRMIESNLRLVVKIARRYGNRGLALLDLI
FSL R95273   YLGEIGYSPLLTAEEEVYFARRALRGDVASRRRMIESNLRLVVKIARRYGNRGLALLDLI
FSL R95274   YLGEIGYSPLLTAEEEVYFARRALRGDVASRRRMIESNLRLVVKIARRYGNRGLALLDLI
FSL R95344   YLGEIGYSPLLTAEEEVYFARRALRGDVASRRRMIESNLRLVVKIARRYGNRGLALLDLI
FSL R95400   YLGEIGYSPLLTAEEEVYFARRALRGDVASRRRMIESNLRLVVKIARRYGNRGLALLDLI
FSL R95402   YLGEIGYSPLLTAEEEVYFARRALRGDVASRRRMIESNLRLVVKIARRYGNRGLALLDLI
FSL R95406   YLGEIGYSPLLTAEEEVYFARRALRGDVASRRRMIESNLRLVVKIARRYGNRGLALLDLI
FSL R95409   YLGEIGYSPLLTAEEEVYFARRALRGDVASRRRMIESNLRLVVKIARRYGNRGLALLDLI
FSL R95495   YLGEIGYSPLLTAEEEVYFARRALRGDVASRRRMIESNLRLVVKIARRYGNRGLALLDLI
FSL R95496   YLGEIGYSPLLTAEEEVYFARRALRGDVASRRRMIESNLRLVVKIARRYGNRGLALLDLI
FSL R95497   YLGEIGYSPLLTAEEEVYFARRALRGDVASRRRMIESNLRLVVKIARRYGNRGLALLDLI
FSL R95498   YLGEIGYSPLLTAEEEVYFARRALRGDVASRRRMIESNLRLVVKIARRYGNRGLALLDLI
FSL R95504   YLGEIGYSPLLTAEEEVYFARRALRGDVASRRRMIESNLRLVVKIARRYGNRGLALLDLI
FSL R96567   YLGEIGYSPLLTAEEEVYFARRALRGDVASRRRMIESNLRLVVKIARRYGNRGLALLDLI
FSL R95252   YLGEIGYSPLLTAEEEVYFARRALRGDVASRRRMIESNLRLVVKIARRYGNRGLALLDLI
FSL R95494   YLGEIGYSPLLTAEEEVYFARRALRGDVASRRRMIESNLRLVVKIARRYGNRGLALLNLI
FSL R95499   YLGEIGYSPLLTAEEEVYFARRALRGDVASRRRMIESNLRLVVKIARRYGNRGLALLDLI
FSL R95502   YLGEIGYSPLLTAEEEVYFARRALRGDVASRRRMIESNLRLVEKIARRYGNRGLALLDLI
```

FSL R95505	EEGNLGLIRAVEKFDPERGFRFNIRNLVDSPDNRTGDYEPNPYDSLADSHC*RAERIPAH
FSL R95220	EEGNLGLIRAVEKFDPERGFRFSTYATWWIRQTIERAIMNQTRTIRLPIHIVKELNVYLR
FSL R95250	EEGNLGLIRAVEKFDPERGFRFSTYATWWIRQTIERAIMNQTRTIRLPIHIVKELNVYLR
FSL R95251	EEGNLGLIRAVEKFDPERGFRFSTYATWWIRQTIERAIMNQTRTIRLPIHIVKELNVYLR
FSL R95272	EEGNLGLIRAVEKFDPERGFRFSTYATWWIRQTIERAIMNQTRTIRLPIHIVKELNVYLR
FSL R95273	EEGNLGLIRAVEKFDPERGFRFSTYATWWIRQTIERAIMNQTRTIRLPIHIVKELNVYLR
FSL R95274	EEGNLGLIRAVEKFDPERGFRFSTYATWWIRQTIERAIMNQTRTIRLPIHIVKELNVYLR
FSL R95344	EEGNLGLIRAVEKFDPERGFRFSTYATWWIRQTIERAIMNQTRTIRLPIHIVKELNVYLR
FSL R95400	EEGNLGLIRAVEKFDPERGFRFSTYATWWIRQTIERAIMNQTRTIRLPIHIVKELNVYLR
FSL R95402	EEGNLGLIRAVEKFDPERGFRFSTYATWWIRQTIERAIMNQTRTIRLPIHIVKELNVYLR
FSL R95406	EEGNLGLIRAVEKFDPERGFRFSTYATWWIRQTIERAIMNQTRTIRLPIHIVKELNVYLR
FSL R95409	EEGNLGLIRAVEKFDPERGFRFSTYATWWIRQTIERAIMNQTRTIRLPIHIVKELNVYLR
FSL R95495	EEGNLGLIRAVEKFDPERGFRFSTYATWWIRQTIERAIMNQTRTIRLPIHIVKELNVYLR
FSL R95496	EEGNLGLIRAVEKFDPERGFRFSTYATWWIRQTIERAIMNQTRTIRLPIHIVKELNVYLR
FSL R95497	EEGNLGLIRAVEKFDPERGFRFSTYATWWIRQTIERAIMNQTRTIRLPIHIVKELNVYLR
FSL R95498	EEGNLGLIRAVEKFDPERGFRFSTYATWWIRQTIERAIMNQTRTIRLPIHIVKELNVYLR
FSL R95504	EEGNLGLIRAVEKFDPERGFRFSTYATWWIRQTIERAIMNQTRTIRLPIHIVKELNVYLR
FSL R96567	EEGNLGLIRAVEKFDPERGFRFSTYATWWIRQTIERAIMNQTRTIRLPIHIVKELNVYLR
FSL R95252	EEGNLGLIRAVEKFDPERGFRFSTYATWWIRQTIERAIMNQTRTIRLPIHIVKELNVYLR
FSL R95494	EEGNLGLIRAVEKFDPERGFRFSTYATWWIRQTIERAIMNQTRTIRLPIHIVKELNVYLR
FSL R95499	EEGNLGLIRAVEKFDPERGFRFSTYATWWIRQTIERAIMNQTRTIRLSIHIVKELNVYLR
FSL R95502	EEGNLGLIRAVEKFDPERGFRFSTYATWWIRQTIERAIMNQTRTIRLPIHIVKELNVYLR

FSL R95505	RT*VVA*TGPRTECGRNCRATG*TG**RQPYASSQRAHYLGRHPAGR*FRKSVAGHPGR*
FSL R95220	TARELSHKLDHEPSAEEIAEQLDKPVDDVSRMLRLNERITSVDTPLGGDSEKALLDILAD
FSL R95250	TARELSHKLDHEPSAEEIAEQLDKPVDDVSRMLRLNERITSVDTPLGGDSEKALLDILAD
FSL R95251	TARELSHKLDHEPSAEEIAEQLDKPVDDVSRMLRLNERITSVDTPLGGDSEKALLDILAD
FSL R95272	TARELSHKLDHEPSAEEIAEQLDKPVDDVSRMLRLNERITSVDTPLGGDSEKALLDILAD
FSL R95273	TARELSHKLDHEPSAEEIAEQLDKPVDDVSRMLRLNERITSVDTPLGGDSEKALLDILAD
FSL R95274	TARELSHKLDHEPSAEEIAEQLDKPVDDVSRMLRLNERITSVDTPLGGDSEKALLDILAD
FSL R95344	TARELSHKLDHEPSAEEIAEQLDKPVDDVSRMLRLNERITSVDTPLGGDSEKALLDILAD
FSL R95400	TARELSHKLDHEPSAEEIAEQLDKPVDDVSRMLRLNERITSVDTPLGGDSEKALLDILAD
FSL R95402	TARELSHKLDHEPSAEEIAEQLDKPVDDVSRMLRLNERITSVDTPLGGDSEKALLDILAD
FSL R95406	TARELSHKLDHEPSAEEIAEQLDKPVDDVSRMLRLNERITSVDTPLGGDSEKALLDILAD
FSL R95409	TARELSHKLDHEPSAEEIAEQLDKPVDDVSRMLRLNERITSVDTPLGGDSEKALLDILAD
FSL R95495	TARELSHKLDHEPSAEEIAEQLDKPVDDVSRMLRLNERITSVDTPLGGDSEKALLDILAD
FSL R95496	TARELSHKLDHEPSAEEIAEQLDKPVDDVSRMLRLNERITSVDTPLGGDSEKALLDILAD
FSL R95497	TARELSHKLDHEPSAEEIAEQLDKPVDDVSRMLRLNERITSVDTPLGGDSEKALLDILAD
FSL R95498	TARELSHKLDHEPSAEEIAEQLDKPVDDVSRMLRLNERITSVDTPLGGDSEKALLDILAD
FSL R95504	TARELSHKLDHEPSAEEIAEQLDKPVDDVSRMLRLNERITSVDTPLGGDSEKALLDILAD
FSL R96567	TARELSHKLDHEPSAEEIAEQLDKPVDDVSRMLRLNERITSVDTPLGGDSEKALLDILAD
FSL R95252	TARELSHKLDHEPSAEEIAEQLDKPVDDVSRMLRLNERITSVDTPLGGDSEKALLDILAD
FSL R95494	TARELSHKLDHEPSAEEIAEQLDKPVDDVSRMLRLNERITSVDTPLGGDSEKALLDILAD
FSL R95499	TARELSHKLDHEPSAEEIAEQLDKPVDDVSRMLRLNERITSVDTPLGGDSEKALLDILAD
FSL R95502	TARELSHKLDHEPSAEEIAEQLDKPVDDVSRMLRLNERITSVDTPLGGDSEKALLDILAD

FSL R95505	KRERSGRHHAR*RYETEHRQMVVRTERQTA*SAGAPFRSAGI*SCDTGRCRP*NRSYA*
FSL R95220	EKENGPEDTTQDDDMKQSIVKWLFELNAKQREVLARRFGLLGYEAATLEDVGREIGLTR
FSL R95250	EKENGPEDTTQDDDMKQSIVKWLFELNAKQREVLARRFGLLGYEAATLEDVGREIGLTR
FSL R95251	EKENGPEDTTQDDDMKQSIVKWLFELNAKQREVLARRFGLLGYEAATLEDVGREIGLTR
FSL R95272	EKENGPEDTTQDDDMKQSIVKWLFELNAKQREVLARRFGLLGYEAATLEDVGREIGLTR
FSL R95273	EKENGPEDTTQDDDMKQSIVKWLFELNAKQREVLARRFGLLGYEAATLEDVGREIGLTR
FSL R95274	EKENGPEDTTQDDDMKQSIVKWLFELNAKQREVLARRFGLLGYEAATLEDVGREIGLTR
FSL R95344	EKENGPEDTTQDDDMKQSIVKWLFELNAKQREVLARRFGLLGYEAATLEDVGREIGLTR
FSL R95400	EKENGPEDTTQDDDMKQSIVKWLFELNAKQREVLARRFGLLGYEAATLEDVGREIGLTR
FSL R95402	EKENGPEDTTQDDDMKQSIVKWLFELNAKQREVLARRFGLLGYEAATLEDVGREIGLTR
FSL R95406	EKENGPEDTTQDDDMKQSIVKWLFELNAKQREVLARRFGLLGYEAATLEDVGREIGLTR
FSL R95409	EKENGPEDTTQDDDMKQSIVKWLFELNAKQREVLARRFGLLGYEAATLEDVGREIGLTR
FSL R95495	EKENGPEDTTQDDDMKQSIVKWLFELNAKQREVLARRFGLLGYEAATLEDVGREIGLTR
FSL R95496	EKENGPEDTTQDDDMKQSIVKWLFELNAKQREVLARRFGLLGYEAATLEDVGREIGLTR
FSL R95497	EKENGPEDTTQDDDMKQSIVKWLFELNAKQREVLARRFGLLGYEAATLEDVGREIGLTR
FSL R95498	EKENGPEDTTQDDDMKQSIVKWLFELNAKQREVLARRFGLLGYEAATLEDVGREIGLTR
FSL R95504	EKENGPEDTTQDDDMKQSIVKWLFELNAKQREVLARRFGLLGYEAATLEDVGREIGLTR
FSL R96567	EKENGPEDTTQDDDMKQSIVKWLFELNAKQREVLARRFGLLGYEAATLEDVGREIGLTR
FSL R95252	EKENGPEDTTQDDDMKQSIVKWLFELNAKQREVLARRFGLLGYEAATLEDVGREIGLTR
FSL R95494	EKENGPEDTTQDDDMKQSIVKWLFELNAKQREVLARRFGLLGYEAATLEDVGREIGLTR
FSL R95499	EKENGPEDTTQDDDMKQSIVKWLFELNAKQREVLARRFGLLGYEAATLEDVGREIGLTR
FSL R95502	EKENGPEDTTQDDDMKQSIVKWLFELNAKQREVLARRFGLLGYEAATLEDVGREIGLTR

FSL R95505	ACSSDSG*RPAPSARNSADAGAEYRSAVPRV
FSL R95220	ERVRQIQVEGLRRLREILQTQGLNIEALFRE
FSL R95250	ERVRQIQVEGLRRLREILQTQGLNIEALFRE
FSL R95251	ERVRQIQVEGLRRLREILQTQGLNIEALFRE
FSL R95272	ERVRQIQVEGLRRLREILQTQGLNIEALFRE
FSL R95273	ERVRQIQVEGLRRLREILQTQGLNIEALFRE
FSL R95274	ERVRQIQVEGLRRLREILQTQGLNIEALFRE
FSL R95344	ERVRQIQVEGLRRLREILQTQGLNIEALFRE
FSL R95400	ERVRQIQVEGLRRLREILQTQGLNIEALFRE
FSL R95402	ERVRQIQVEGLRRLREILQTQGLNIEALFRE
FSL R95406	ERVRQIQVEGLRRLREILQTQGLNIEALFRE
FSL R95409	ERVRQIQVEGLRRLREILQTQGLNIEALFRE
FSL R95495	ERVRQIQVEGLRRLREILQTQGLNIEALFRE
FSL R95496	ERVRQIQVEGLRRLREILQTQGLNIEALFRE
FSL R95497	ERVRQIQVEGLRRLREILQTQGLNIEALFRE
FSL R95498	ERVRQIQVEGLRRLREILQTQGLNIEALFRE
FSL R95504	ERVRQIQVEGLRRLREILQTQGLNIEALFRE
FSL R96567	ERVRQIQVEGLRRLREILQTQGLNIEALFRE
FSL R95252	ERVRQIQVEGLRRLREILQTQGLNIEALFRE
FSL R95494	ERVRQIQVEGLRRLREILQTQGLNIEALFRE
FSL R95499	ERVRQIQVEGLRRLREILQTQGLNIEALFRE
FSL R95502	ERVRQIQVEGLRRLREILQTQGLNIEALFRE
