# Supplementary material for: Assembly and Characterization of a Pathogen Strain Collection for Produce Safety Applications: Pre-growth Conditions Have a Larger Effect on Peroxyacetic Acid Tolerance Than Strain Diversity
Source: Front Microbiol. 2019 May 31;10:1223. doi: 10.3389/fmicb.2019.01223 (PMC6558390; doi:10.3389/fmicb.2019.01223)
Supplement: Supplementary file 7 [file Data_Sheet_6.PDF]

S Figure 6: Muscle alignment of rpoS amino acid sequences of *E. coli*

```
              1        10        20        30        40        50        60
              |        |         |         |         |         |         |
FSL R9-5512   MSQNTLKVHDLNEDAEFDENGVEVFDEKALVEEEPSDNDLAEEELLSQGATQRVLDATQL
FSL R9-5257   MSQNTLKVHDLNEDAEFDENGVEVFDEKALVEEEPSDNDLAEEELLSQGATQRVLDATQL
FSL R9-5513   MSQNTLKVHDLNEDAEFDENGVEVFDEKALVEEEPSDNDLAEEELLSQGATQRVLDATQL
FSL R9-3467   MSQNTLKVHDLNEDAEFDENGVEVLTKRP**KRNPVITIWPKRNCYRREPHSVCWTRLSF
FSL R9-4077   MSQNTLKVHDLNEDAEFDENGVEVFDEKALVEEEPSDNDLAEEELLSQGATQRVLDATQL
FSL R9-4078   MSQNTLKVHDLNEDAEFDENGVEVFDEKALVEEEPSDNDLAEEELLSQGATQRVLDATQL
FSL R9-4079   MSQNTLKVHDLNEDAEFDENGVEVFDEKALVEEEPSDNDLAEEELLSQGATQRVLDATQL
FSL R9-5256   MSQNTLKVHDLNEDAEFDENGVEVFDEKALVEEEPSDNDLAEEELLSQGATQRVLDATQL
FSL R9-5258   MSQNTLKVHDLNEDAEFDENGVEVFDEKALVEEEPSDNDLAEEELLSQGATQRVLDATQL
FSL R9-5271   MSQNTLKVHDLNEDAEFDENGVEVFDEKALVEEEPSDNDLAEEELLSQGATQRVLDATQL
FSL R9-5345   MSQNTLKVHDLNEDAEFDENGVEVFDEKALVEEEPSDNDLAEEELLSQGATQRVLDATQL
FSL R9-5509   MSQNTLKVHDLNEDAEFDENGVEVFDEKALVEEEPSDNDLAEEELLSQGATQRVLDATQL
FSL R9-5515   MSQNTLKVHDLNEDAEFDENGVEVFDEKALVEEEPSDNDLAEEELLSQGATQRVLDATQL
FSL R9-5517   MSQNTLKVHDLNEDAEFDENGVEVFDEKALVEEEPSDNDLAEEELLSQGATQRVLDATQL
FSL R9-5639   MSQNTLKVHDLNEDAEFDENGVEVFDEKALVEEEPSDNDLAEEELLSQGATQRVLDATQL
FSL R9-6071   MSQNTLKVHDLNEDAEFDENGVEVFDEKALVEEEPSDNDLAEEELLSQGATQRVLDATQL

FSL R9-5512   YLGEIGYSPLLTAEEEVYFARRALRGDVASRRRMIESNLRLVVKIARRYGNRGLALLDLI
FSL R9-5257   YLGEIGYSPLLTAEEEVYFARRALRGDVASRRRMIESNLRLVVKIARRYGNRGLALLNLI
FSL R9-5513   YLGEIGYSPLLTAEEEVYFARRALRGDVASRRRMIESNLRLVVKIARRYGNRGLALLDLI
FSL R9-3467   TLVRLVIHHC*RPKKKFILRVAHCVEMSPLAAG*SRVTCVWW*KLPAVMAIVVWRCWTLS
FSL R9-4077   YLGEIGYSPLLTAEEEVYFARRALRGDVASRRRMIESNLRLVVKIARRYGNRGLALLDLI
FSL R9-4078   YLGEIGYSPLLTAEEEVYFARRALRGDVASRRRMIESNLRLVVKIARRYGNRGLALLDLI
FSL R9-4079   YLGEIGYSPLLTAEEEVYFARRALRGDVASRRRMIESNLRLVVKIARRYGNRGLALLDLI
FSL R9-5256   YLGEIGYSPLLTAEEEVYFARRALRGDVASRRRMIESNLRLVVKIARRYGNRGLALLDLI
FSL R9-5258   YLGEIGYSPLLTAEEEVYFARRALRGDVASRRRMIESNLRLVVKIARRYGNRGLALLDLI
FSL R9-5271   YLGEIGYSPLLTAEEEVYFARRALRGDVASRRRMIESNLRLVVKIARRYGNRGLALLDLI
FSL R9-5345   YLGEIGYSPLLTAEEEVYFARRALRGDVASRRRMIESNLRLVVKIARRYGNRGLALLDLI
FSL R9-5509   YLGEIGYSPLLTAEEEVYFARRALRGDVASRRRMIESNLRLVVKIARRYGNRGLALLDLI
FSL R9-5515   YLGEIGYSPLLTAEEEVYFARRALRGDVASRRRMIESNLRLVVKIARRYGNRGLALLDLI
FSL R9-5517   YLGEIGYSPLLTAEEEVYFARRALRGDVASRRRMIESNLRLVVKIARRYGNRGLALLDLI
FSL R9-5639   YLGEIGYSPLLTAEEEVYFARRALRGDVASRRRMIESNLRLVVKIARRYGNRGLALLDLI
FSL R9-6071   YLGEIGYSPLLTAEEEVYFARRALRGDVASRRRMIESNLRLVVKIARRYGNRGLALLDLI

FSL R9-5512   EEGNLGLIRAVEKFDPERGFRFSTYATWWIRQTIERAIMNQTRTIRLPIHIVKELNVYLR
FSL R9-5257   EEGNLGLIRAVEKFDPERGFRFSTYATWWIRQTIERAIMNQTRTIRLPIHIVKELNVYLR
FSL R9-5513   EEGNLGLIRAVEKFDPERGFRFSTYATWWIRQTIERAIMNQTRTIRLPIHIVKELNVYLR
FSL R9-3467   KRATWG*SVR*RSLTRNVVSASQHTQPGGFARRLNGRL*TKPVLFVCRFTS*RS*TFTCE
FSL R9-4077   EEGNLGLIRAVEKFDPERGFRFSTYATWWIRQTIERAIMNQTRTIRLPIHIVKELNVYLR
FSL R9-4078   EEGNLGLIRAVEKFDPERGFRFSTYATWWIRQTIERAIMNQTRTIRLPIHIVKELNVYLR
FSL R9-4079   EEGNLGLIRAVEKFDPERGFRFSTYATWWIRQTIERAIMNQTRTIRLPIHIVKELNVYLR
FSL R9-5256   EEGNLGLIRAVEKFDPERGFRFSTYATWWIRQTIERAIMNQTRTIRLPIHIVKELNVYLR
FSL R9-5258   EEGNLGLIRAVEKFDPERGFRFSTYATWWIRQTIERAIMNQTRTIRLPIHIVKELNVYLR
FSL R9-5271   EEGNLGLIRAVEKFDPERGFRFSTYATWWIRQTIERAIMNQTRTIRLPIHIVKELNVYLR
FSL R9-5345   EEGNLGLIRAVEKFDPERGFRFSTYATWWIRQTIERAIMNQTRTIRLPIHIVKELNVYLR
FSL R9-5509   EEGNLGLIRAVEKFDPERGFRFSTYATWWIRQTIERAIMNQTRTIRLPIHIVKELNVYLR
FSL R9-5515   EEGNLGLIRAVEKFDPERGFRFSTYATWWIRQTIERAIMNQTRTIRLPIHIVKELNVYLR
FSL R9-5517   EEGNLGLIRAVEKFDPERGFRFSTYATWWIRQTIERAIMNQTRTIRLPIHIVKELNVYLR
FSL R9-5639   EEGNLGLIRAVEKFDPERGFRFSTYATWWIRQTIERAIMNQTRTIRLPIHIVKELNVYLR
FSL R9-6071   EEGNLGLIRAVEKFDPERGFRFSTYATWWIRQTIERAIMNQTRTIRLPIHIVKELNVYLR
```

```
FSL R9-5512   TARELSHKLDHEPSAEEIAEQLDKPVDDVSRMLRLNERITSVDTPLGGDSEKALLDILAD
FSL R9-5257   TARELSHKLDHEPSAEEIAEQLDKPVDDVSRMLRLNERITSVDTPLGGDSEKALLDILAD
FSL R9-5513   TARELSHKLDHEPSAEEIAEQLDKPVDDVSRMLRLNERITSVDTPLGGDSEKALLDILAD
FSL R9-3467   PHVSCPISWTMNQVRKRSQSNWISQLMTSAVCFVLTSALPR*TPRWVVIPKKRCWTSWPM
FSL R9-4077   TARELSHKLDHEPSAEEIAEQLDKPVDDVSRMLRLNERITSVDTPLGGDSEKALLDILAD
FSL R9-4078   TARELSHKLDHEPSAEEIAEQLDKPVDDVSRMLRLNERITSVDTPLGGDSEKALLDILAD
FSL R9-4079   TARELSHKLDHEPSAEEIAEQLDKPVDDVSRMLRLNERITSVDTPLGGDSEKALLDILAD
FSL R9-5256   TARELSHKLDHEPSAEEIAEQLDKPVDDVSRMLRLNERITSVDTPLGGDSEKALLDILAD
FSL R9-5258   TARELSHKLDHEPSAEEIAEQLDKPVDDVSRMLRLNERITSVDTPLGGDSEKALLDILAD
FSL R9-5271   TARELSHKLDHEPSAEEIAEQLDKPVDDVSRMLRLNERITSVDTPLGGDSEKALLDILAD
FSL R9-5345   TARELSHKLDHEPSAEEIAEQLDKPVDDVSRMLRLNERITSVDTPLGGDSEKALLDILAD
FSL R9-5509   TARELSHKLDHEPSAEEIAEQLDKPVDDVSRMLRLNERITSVDTPLGGDSEKALLDILAD
FSL R9-5515   TARELSHKLDHEPSAEEIAEQLDKPVDDVSRMLRLNERITSVDTPLGGDSEKALLDILAD
FSL R9-5517   TARELSHKLDHEPSAEEIAEQLDKPVDDVSRMLRLNERITSVDTPLGGDSEKALLDILAD
FSL R9-5639   TARELSHKLDHEPSAEEIAEQLDKPVDDVSRMLRLNERITSVDTPLGGDSEKALLDILAD
FSL R9-6071   TARELSHKLDHEPSAEEIAEQLDKPVDDVSRMLRLNERITSVDTPLGGDSEKALLDILAD

FSL R9-5512   EKENGPEDTTQDDDMKQSIVKWLFELNAKQREVLARRFGLLGYEAATLEDVGREIGLTRE
FSL R9-5257   EKENGPEDTTQDDDMKQSIVKWLFELNAKQREVLARRFGLLGYEAATLEDVGREIGLTRE
FSL R9-5513   EKENGPEDTTQDDDMKQSIVKWLFELNAKQREVLARRFGLLGYEAATLEDVGREIGLTRE
FSL R9-3467   KKRTVRKIPRKMTI*SRASSNGCSS*TPNSVKYWHVDSVCWGTKRQHWKM*VVKLASPVN
FSL R9-4077   EKENGPEDTTQDDDMKQSIVKWLFELNAKQREVLARRFGLLGYEAATLEDVGREIGLTRE
FSL R9-4078   EKENGPEDTTQDDDMKQSIVKWLFELNAKQREVLARRFGLLGYEAATLEDVGREIGLTRE
FSL R9-4079   EKENGPEDTTQDDDMKQSIVKWLFELNAKQREVLARRFGLLGYEAATLEDVGREIGLTRE
FSL R9-5256   EKENGPEDTTQDDDMKQSIVKWLFELNAKQREVLARRFGLLGYEAATLEDVGREIGLTRE
FSL R9-5258   EKENGPEDTTQDDDMKQSIVKWLFELNAKQREVLARRFGLLGYEAATLEDVGREIGLTRE
FSL R9-5271   EKENGPEDTTQDDDMKQSIVKWLFELNAKQREVLARRFGLLGYEAATLEDVGREIGLTRE
FSL R9-5345   EKENGPEDTTQDDDMKQSIVKWLFELNAKQREVLARRFGLLGYEAATLEDVGREIGLTRE
FSL R9-5509   EKENGPEDTTQDDDMKQSIVKWLFELNAKQREVLARRFGLLGYEAATLEDVGREIGLTRE
FSL R9-5515   EKENGPEDTTQDDDMKQSIVKWLFELNAKQREVLARRFGLLGYEAATLEDVGREIGLTRE
FSL R9-5517   EKENGPEDTTQDDDMKQSIVKWLFELNAKQREVLARRFGLLGYEAATLEDVGREIGLTRE
FSL R9-5639   EKENGPEDTTQDDDMKQSIVKWLFELNAKQREVLARRFGLLGYEAATLEDVGREIGLTRE
FSL R9-6071   EKENGPEDTTQDDDMKQSIVKWLFELNAKQREVLARRFGLLGYEAATLEDVGREIGLTRE

FSL R9-5512   RVRQIQVEGLRRLREILQTQGLAVPRVSKH
FSL R9-5257   RVRQIQVEGLRRLREILQTQGLNIEALFRE
FSL R9-5513   RVRQIQVEDLRRLREILQTQGLNIEALFRE
FSL R9-3467   VFARFRLKACAVCAKSCKRRG*ISKRCSAS
FSL R9-4077   RVRQIQVEGLRRLREILQTQGLNIEALFRE
FSL R9-4078   RVRQIQVEGLRRLREILQTQGLNIEALFRE
FSL R9-4079   RVRQIQVEGLRRLREILQTQGLNIEALFRE
FSL R9-5256   RVRQIQVEGLRRLREILQTQGLNIEALFRE
FSL R9-5258   RVRQIQVEGLRRLREILQTQGLNIEALFRE
FSL R9-5271   RVRQIQVEGLRRLREILQTQGLNIEALFRE
FSL R9-5345   RVRQIQVEGLRRLREILQTQGLNIEALFRE
FSL R9-5509   RVRQIQVEGLRRLREILQTQGLNIEALFRE
FSL R9-5515   RVRQIQVEGLRRLREILQTQGLNIEALFRE
FSL R9-5517   RVRQIQVEGLRRLREILQTQGLNIEALFRE
FSL R9-5639   RVRQIQVEGLRRLREILQTQGLNIEALFRE
FSL R9-6071   RVRQIQVEGLRRLREILQTQGLNIEALFRE
```
