# Supplementary material for: Assembly and Characterization of a Pathogen Strain Collection for Produce Safety Applications: Pre-growth Conditions Have a Larger Effect on Peroxyacetic Acid Tolerance Than Strain Diversity
Source: Front Microbiol. 2019 May 31;10:1223. doi: 10.3389/fmicb.2019.01223 (PMC6558390; doi:10.3389/fmicb.2019.01223)
Supplement: Supplementary file 8 [file Data_Sheet_7.PDF]

Supplemental table 1: Initially proposed strain collection for evaluation by experts

Please rank based on scale:
1-strain is irrelevant, do not include
2-strain may be relevant, but probably not
3-uncertain, strain may or may not be relevant
4-important, should be included
5-very important, must be included

Genus	Species	Lineage	Serotype	Isolate ID number	Isolate origin and reason for inclusion	Reference
			**Proposed *Salmonella enterica* strains**			
Salmonella	*enterica*		Saintpaul	*to be acquired**	Jalepeno peppers, 2008, multistate US and Canada	MMWR August 29, 2008 / 57(34);929-934
			Tennessee	*to be acquired**	Peanut butter, 2006-7, multistate US	MMWR June 1, 2007 / 56(21);521-524
			Typhimurium	*to be acquired**	Orange Juice, 2005, multistate US	Jain et al., Clinical Infectious Diseases 2009;48:1065–1071
			Poona	*to be acquired**	Cantaloupe, 2000-2, US and Canada	MMWR November 22, 2002 / 51(46);1044-1047
			PT30	Available through ATCC (BAA 1045)	Almonds, 2000-1, US and Canada	Isaacs et al., JFP, Vol. 68, No. 1, 2005, Pages 191–198
			Javiana	*to be acquired**	Tomatoes, 2002, multistate US	Srikantiah et al. Emerg Infect Dis. 2005 March
			Stanley	*to be acquired**	Alfalfa Sprouts, 1995, US and Finland	Mahon et al., J Infect Dis. 1997 Apr;175(4):876-82
			Newport (antimicrobial susceptible)	*to be acquired**	Tomatoes, 2002 and 2005, multistate US	S. K. Greene,et al. 2005. Epidemiology and Infection, 136, pp 157-165
			Senftenberg 775W	Available through ATCC (43845)	Heat resistant Seftenberg, used for validation studies	
			Hartford	*to be acquired**	Organic sprouted chia seed powder, USA 2014	http://www.cdc.gov August 13, 2014 2:30 PM ET
			Heidelberg	*to be acquired**	Poultry Producer, 2012-2013, multistate USA	MMWR July 12, 2013 / 62(27);553-556
			Typhimurium	*to be acquired**	Peanut butter, 2008-2009, multistate US	MMWR January 29, 2009 / 58 (Early Release);1-6
			Chester	*to be acquired**	Frozen meals, 2010, multistate US	MMWR December 6, 2013 / 62(48);979-982
			Bovismorbificans	*to be acquired**	Hummus and Tahini, 2011, multistate US	MMWR November 23, 2012 / 61(46);944-947
			I 4,[5],12:i:-	*to be acquired**	Alfalfa Sprouts, 2010-2011, multistate US	www.cdc.gov February 10, 2011
			Montevideo	*to be acquired**	Pistacio nuts, 2009, US	www.cdc.gov April 14, 2009
			Litchfield	*to be acquired**	Cantaloupe, 2009, multistate US	www.cdc.gov April 2, 2008
			Wandsworth	*to be acquired**	Veggie Booty, 2007, multistate US	www.cdc.gov July 18, 2007
			Poona	*to be acquired**	Cucumbers, 2015, multistate US	www.cdc.gov January 26, 2016 2:30 PM ET
			Breanderup	*to be acquired**	nut butter, 2014, multistate US	www.fda.gov October 17, 2014
			Paratyphi B	*to be acquired**	Frozen Raw Tuna, 2015, multistate US	www.cdc.gov August 19, 2015 5:30 PM ET
			Newport	*to be acquired**	Cucumbers, 2014, multistate US	MMWR February 20, 2015 / 64(06);144-147
			Newport (MDR)	*to be acquired**	Undercooked ground beef, 2002, multistate US (included for validation study with intervention treatments)	MMWR June 28, 2002 / 51(25);545-548
			Enteriditis	*to be acquired**	Bean sprouts, 2014, multistate US	www.cdc.gov January 23, 2015 5:00 PM ET
			**Proposed *Listeria monocytogenes* strains**			
Listeria	*monocytogenes*	I	4b	FSL J1-108	Coleslaw, human, epidemic, Halifax, 1981 (included to assure representation of a serotype 4b isolate)	
		I	1/2b	FSL R2-503	Human, epidemic, Illinois (1994) (included to assure representation of a serotype 1/2b isolate)	
		I	4d	FSL J1-107	Human epidemic, coleslaw (included to assure representation of a serotype 4d isolate)	
		II	1/2a	FSL J1-101	Hot dog, human, sporadic, US (included to assure representation of a serotype 1/2a isolate)	
		not available		*to be acquired**	Caramel Apple, Dec 2014-2015, multistate US	www.cdc.gov February 12, 2015 4:30 PM ET

		not available		*to be acquired**	Packaged Salad Dole, 2016, multistate US	www.cdc.gov February 25, 2016 2:00 PM ET
		not available		*to be acquired**	Sprouts from Wholesome, 2014, Illinois, Michigan	www.cdc.gov January 27, 2015 10:30 AM ET
		III	4a	FSL J1-031	Human sporadic case (included to assure representation of a lineage III isolate)	
		IV	4b	FSL J1-158	Animal, goat (included to assure representation of a lineage IV isolate)	
		II	*not available*	FSL S10-2161	Soil, spinach field	Appl Environ Microbiol 81(17): 6059-6069.
		II	*not available*	FSL S10-2371	Soil, spinach field	Appl Environ Microbiol 81(17): 6059-6069.
		I	*not available*	FSL S10-2177	Soil, spinach field	Appl Environ Microbiol 81(17): 6059-6069.
		I	*not available*	FSL S10-2262	Soil, spinach field	Appl Environ Microbiol 81(17): 6059-6069.
**Proposed *Escherichia coli* strains**						
Escherichia	*coli*		O26	*to be acquired**	Chiptole Mexican Grill, 2015, multistate US (included to assure representation of serotypes of "big six")	www.cdc.gov February 1, 2016 12:00 PM ET
			O121	*to be acquired**	Raw Clover Sprouts, 2014, multistate US (included to assure representation of serotypes of "big six")	www.cdc.gov August 1, 2014 2:15 PM ET
			O121	*to be acquired**	Farm Rich Brand Frozen Food Products, 2013, multistate US (included to assure representation of serotypes of "big six")	www.cdc.gov May 30, 2013 01:00 PM ET
			O145	*to be acquired**	Multistate Outbreak, 2012 (included to assure representation of serotypes of "big six")	www.cdc.gov July 20, 2012 3:30 PM ET
			O26	*to be acquired**	Raw Clover Sprouts, 2012, multistate US (included to assure representation of serotypes of "big six")	www.cdc.gov April 3, 2012 4:45 PM ET
			O157	*to be acquired**	Alfalfa Sprouts, 2016, Minnesota and Wisconsin	http://www.cdc.gov March 2, 2016 10:30 AM ET
			O111:H8	*to be acquired**	Outbreak among teenage campers, 2012, Texas (included to assure representation of serotypes of "big six")	MMWR April 21, 2000 / 49(15);321-4
			O111	*to be acquired**	Facility Dairy, 2010, Coloardo (included to assure representation of serotypes of "big six")	MMWR March 9, 2012 / 61(09);149-152
			O45:H2	*to be acquired**	Isolates from human cases (included to assure representation of serotypes of "big six")	Genome Announc. 2014 Jul 10;2(4). pii: e00501-14. doi: 10.1128/genomeA.00501-14.
			O103	*to be acquired**	Isolates from human cases (included to assure representation of serotypes of "big six")	Foodborne Pathog Dis. 2013 May;10(5):453-60. doi: 10.1089/fpd.2012.1401. Epub 2013 Apr 6.
**Proposed index, indicator and surrogate strains**						
Listeria	*innocua*	*not available*		FSL C2-0008	Fish processing plant, sampled 2000 (surrogate for *Listeria monocytogenes)*	
Listeria	*innocua* (hly+)	III	4b	FSL J1-0023	Unkown (donor Qualicon) (surrogate for *Listeria monocytogenes*)	
Listeria	*marthii*	*not available*		FSL C7-0084	Connecticut Hill Wildlife Management Area, NY - environment, non-food 2009 (surrogate for *Listeria monocytogenes*)	Appl. Environ. Microbiol. January 2013 vol. 79 no. 2 588-600
Escherichia	*coli*			FSL R9-4077	Generic *E. coli* , environmental water, plant and soil isolate (surrogate organism for *E. coli* O157:H7, used in survival study on leafy greens)	Int J Food Microbiol. 2011 Dec 2;151(2):216-22. doi: 10.1016/j.ijfoodmicro.2011.08.027. Epub 2011 Sep 2.

Escherichia	*coli*	FSL R9-4078	Generic *E. coli* , environmental water, plant and soil isolate (surrogate organism for E. coli O157:H7, used in survival study on leafy greens)	Int J Food Microbiol. 2011 Dec 2;151(2):216-22. doi: 10.1016/j.ijfoodmicro.2011.08.027. Epub 2011 Sep 2.
Escherichia	*coli*	FSL R9-4079	Generic *E. coli* , environmental water, plant and soil isolate (surrogate organism for E. coli O157:H7, used in survival study on leafy greens)	Int J Food Microbiol. 2011 Dec 2;151(2):216-22. doi: 10.1016/j.ijfoodmicro.2011.08.027. Epub 2011 Sep 2.
Escherichia	*coli*	ATCC 700728<br>FSL R9-3467	Naturally occurring non-pathogenic *E. coli* (attenuated STEC O157:H7)	J Food Prot. 2015 Feb;78(2):240-7. doi: 10.4315/0362-028X.JFP-14-277.
Escherichia	*coli*	P3 - BAA1428	Isolated from cattle (nonpathogenic *E.coli* surrogate for *Salmonella*)	Journal of Food Protection, Number 4, April 2008, pp. 676-873, pp. 714-718(5)
Enterococcus	*faecalis*	ATCC 29212		
Enterococcus	*faecium*	NRRL B-2354		

Further information for listed strains with 'FSL' ID can be found in the bacterial strain database: Food Microbe Tracker

Suggestion for additional strains:
