# Supplementary material for: Assembly and Characterization of a Pathogen Strain Collection for Produce Safety Applications: Pre-growth Conditions Have a Larger Effect on Peroxyacetic Acid Tolerance Than Strain Diversity
Source: Front Microbiol. 2019 May 31;10:1223. doi: 10.3389/fmicb.2019.01223 (PMC6558390; doi:10.3389/fmicb.2019.01223)
Supplement: Supplementary file 9 [file Data_Sheet_8.PDF]

Supplemental table 2: Pre-growth conditions

**Pre-growth condition**	**Medium**	**Target OD *Salmonella enterica***	**Target OD *E. coli***	**Target OD *Listeria***
**Mid-log phase**	100mL TSB	3h post inoculation	OD 0.3	OD 0.4
**Early tationary phase**	100mL TSB	OD 0.2 plus 3h	OD 0.3 plus 3h	OD 0.4 plus 3h
**High salt**	100mL TSB, addition of 4% NaCl	OD 0.6 plus 3h	OD 0.3 plus 3h	OD 0.2 plus 3h
**Low pH**	100mL TSB, addition of Lactic acetic acid: pH 5.0 (*Salmonella*, *E.* coli), pH 5.5 (*Listeria*)	OD 0.15 plus 3h	OD 0.15 plus 3h (FSL R9-5515)<br>OD 0.3 plus 3h (FSL R9-5517, FSL R9-5516, FSL R9-5258, FSL R9-4077, FSL R9-5271)	OD 0.2 plus 3h
**Low water activity**	100mL TSB, addition of glycerol: 0.95 (*Listeria*), 0.96 (*E. coli*, *Salmonella*)	OD 0.5 plus 3h	OD 0.2 plus 3h (FSL R9-5516)<br>OD 0.3 plus 3h (FSL R9-5517, FSL R9-5515, FSL R9-5258, FSL R9-4077, FSL R9-5271)	OD 0.2 plus 3h (FSLR9-5411)<br>OD 0.4 plus 3h (FSL R9-5506, FSL R9-0506, FSL J1-0031)<br>OD 0.5 plus 3h (FSL C2-0008)
**21°C**	100mL TSB	OD 0.6 plus 3h	OD 0.4 plus 3h (FSL R9 -4077, FSL R9-5271)<br>OD 0.6 (FSL R9-5517, FSL R9-5515, FSL R9-5258, FSL R9-5516)	OD 0.6 plus 3h
**Minimal medium**	100mL M9 minimal medium (*Salmonella*, *E. coli*),<br>30mL defined medium (*Listeria*)	OD 0.3 plus 3h	OD 0.2 plus 3h (FSL R9-5271)<br>OD 0.4 plus 3h (FSL R9-5517, FSL R9-5515, FSL R9-5258, FSL R9-5516)<br>OD 0.5 plus 3h (FSL R9-4077)	OD 0.4 plus 3h (FSL R9-0506, FSL F1-0031, FSL R9-5411, FSL R9-5506)<br>OD 0.5 plus 3h (FSL C2-0008)
