# Supplementary material for: Assembly and Characterization of a Pathogen Strain Collection for Produce Safety Applications: Pre-growth Conditions Have a Larger Effect on Peroxyacetic Acid Tolerance Than Strain Diversity
Source: Front Microbiol. 2019 May 31;10:1223. doi: 10.3389/fmicb.2019.01223 (PMC6558390; doi:10.3389/fmicb.2019.01223)
Supplement: Supplementary file 10 [file Data_Sheet_9.PDF]

Supplemental Table 3: Strain requests for strains provided by the FDA, USDA, ATCC or the STEC Center

Serotype	FSL ID	Previous ID	Isolate source (* indicates outbreak associated sources)	Contact (Institute); email or website
Salmonella enterica				
Saintpaul	R9-5400	CFSAN004126	Jalepeno peppers, 2008*	Anna Maounounen-Laasri (FDA); anna.laasri@fda.hhs.gov
Tennessee	R9-5402	CFSAN001371	Peanut butter, 2006-2007*	Anna Maounounen-Laasri (FDA); anna.laasri@fda.hhs.gov
Typhimurium	R9-5409	CFSAN016159	Peanut butter, 2008-2009*	Anna Maounounen-Laasri (FDA); anna.laasri@fda.hhs.gov
Enteritidis - PT30	R9-5272	ATCC BAA 1045	Almonds, 2000-2001	(ATCC); www.atcc.org
Javiana	R9-5273	ATCC BAA-1593	Tomatoes, 2002*	(ATCC); www.atcc.org
Senftenberg 775W	R9-5274	ATCC 43845	Chinese egg powder, 1941*	(ATCC); www.atcc.org
Montevideo	R9-5406	531954	--	Anna Maounounen-Laasri (FDA); anna.laasri@fda.hhs.gov
Listeria monocytogenes				
1/2 b	R9-5411	897760	Caramel apple, 2015	Anna Maounounen-Laasri (FDA); anna.laasri@fda.hhs.gov
Escherichia coli				
O104:H4	R9-5256	2011C-3493	Sprouts, Germany, 2011*	Pina Fratamico (USDA); pina.fratamico@ars.usda.gov
O104:H4	R9-5257	2009EL-2071	Human, Republic of Georgia, 2009	Pina Fratamico (USDA); pina.fratamico@ars.usda.gov
O104:H4	R9-5258	2009EL-2050	Human, Republic of Georgia, 2009	Pina Fratamico (USDA); pina.fratamico@ars.usda.gov
O26:H11	R9-5639	TW016501	Sprout, 2012*	Rebekah Mosci (STEC Center); stec@cvm.msu.edu
Surrogate organisms				
E. coli O157:H7	R9-3467	ATCC 700728	Naturally occurring non-pathogenic *E. coli*	(ATCC); www.atcc.org
E. faecium	R9-5275	ATCC 8459	Salmonella Surrogate (*E. faecium*)	(ATCC); www.atcc.org

-- Indicates that information is not available
