# Supplementary material for: Assembly and Characterization of a Pathogen Strain Collection for Produce Safety Applications: Pre-growth Conditions Have a Larger Effect on Peroxyacetic Acid Tolerance Than Strain Diversity
Source: Front Microbiol. 2019 May 31;10:1223. doi: 10.3389/fmicb.2019.01223 (PMC6558390; doi:10.3389/fmicb.2019.01223)
Supplement: Supplementary file 11 [file Data_Sheet_11.PDF]

Supplemental table 5: Non-synonymous mutations in antibiotic resistant *Salmonella* strains

**FSL ID**	**No. of SNP**	**Position**	**Strand**	**Gene**	**Amino acid change**
FSL R9-6567	2	NODE 12 (pos. 68512)	+	*flhE*	Leu -> Ser
		NODE 54 (pos. 6996)	-	*rpoB*	Arg -> His
FSL R9-5220	1	NODE 6 (pos. 364027)	+	*gyrA*	Ser -> Stop
FSL R9-5251	9	NODE 4 (pos. 145900)	-	*fadJ*	Leu -> Pro
		NODE 6 (pos. 288419)	+	*fadR*	Val -> Ile
		NODE 6 (pos. 319958)	-	hypothetical protein	Ile -> Val
		NODE 9 (pos. 51000)	+	*hemL*	Pro->Thr
		NODE 11 (pos. 52140)	-	*glpR*	Asn -> Lys
		NODE 13 (pos. 109858)	+	hypothetical protein	Ile -> Thr
		NODE 19 (pos. 25052)	-	*yhdA*	Stop -> Tyr
		NODE 20 (pos. 19872)	-	*rpoC*	Arg -> Cys
		NODE 20 (pos. 21554)	-	*rpoC*	Val -> Ala
