# Supplementary material for: Assembly and Characterization of a Pathogen Strain Collection for Produce Safety Applications: Pre-growth Conditions Have a Larger Effect on Peroxyacetic Acid Tolerance Than Strain Diversity
Source: Front Microbiol. 2019 May 31;10:1223. doi: 10.3389/fmicb.2019.01223 (PMC6558390; doi:10.3389/fmicb.2019.01223)
Supplement: Supplementary file 12 [file Data_Sheet_12.PDF]

Supplemental table 6: Mean log reduction, standard deviation and number of trials below detection limit after PAA exposure for strains and conditions

	Mean log reduction after PAA exposure for cells pre-grown at [a]							Arithmetic mean across conditions
	21°C	High salt	Low pH	Reduced water activity	Minimal medium	Mid-log phase	Stationary phase	
Listeria monocytogenes	2.5 – 4.6 (3.3)	0.5 – 1.8 (1.0)	1.6 – 2.6 (2.1)	2.0 – 3.8 (3.0)	4.5 – 6.4 (5.6)	1.8 – 3.9 (3.1)	0.9 – 2.8 (1.8)	
FSL J1-031	3.3 ±1.5	0.5 ±0.2	1.7 ±0.4	2.0 ±0.8	4.5 ±1.6	1.8 ±1.1	0.9 ±0.1	2.1
FSL R9-5506	2.7 ±2.4	0.8 ±0.1	1.6 ±1.3	3.6 ±0.9	5.6 ±0.8	2.9 ±0.3	2.8 ±0.9	2.9
FSL R9-5411	2.5 ±3.0	1.8 ±0.7	2.3 ±1.3	3.8 ±1.5	6.4 ±0.9[2]	3.9 ±0.8	1.0 ±0.1	3.1
FSL R9-0506	4.6 ±2.4[1]	0.9 ±0.2	2.6 ±2.1	2.7 ±1.2	5.9 ±1.1[1]	3.7 ±0.1	2.6 ±0.9	3.3
FSL C2-0008 [b]	4.2 ±0.5	0.7 ±0.2	1.9 ±1.0	1.5 ±0.7	4.9 ±2.2	2.5 ±0.2	2.9 ±0.8	2.7
Salmonella enterica	4.8 – 6.8 (5.8)	2.6 – 4.3 (3.1)	4.9 – 6.4 (5.9)	4.5 – 6.6 (5.4)	4.8 – 7.1 (5.5)	6.0 – 6.0 (6.0)	4.2 – 6.3 (5.3)	
FSL R9-5400	6.7 ±0.6 [2]	2.7 ±1.0	6.0 ±0.9	5.2 ±1.6 [1]	5.2 ±1.7 [2]	6.0 ±0.1 [2]	5.4 ±0.6	5.3
FSL R9-5272	6.8 ±0.5 [2]	4.3 ±2.2	6.4 ±0.4 [1]	6.6 ±0.9 [2]	7.1 ±0.3 [2]	6.0 ±0.0 [1]	6.3 ±0.8 [1]	6.2
FSL R9-5344	4.8 ±0.8	2.6 ±0.8	6.2 ±0.5 [1]	5.2 ±0.5	4.8 ±1.8	6.0 ±0.1 [3]	5.5 ±0.8	5.0
FSL R9-5502	4.9 ±0.5	2.8 ±0.6	4.9 ±0.5	4.5 ±0.7	5.1 ±1.9 [1]	6.0 ±0.2 [3]	4.2 ±0.6	4.6
FSL R9-5275 [b]	0.0 ±0.1	0.0 ±0.1	0.1 ±0.1	0.6 ±0.4	-	3.9 ±1.5 [1]	0.3 ±0.6	0.8
FSL R9-6232 [b]	4.0 ±1.0	5.4 ±1.3 [1]	4.4 ±0.8	5.9 ±0.9 [1]	6.8 ±0.8 [2]	6.0 ±0.1 [3]	4.2 ±0.7	5.3
STEC	2.0 – 3.9 (2.9)	1.5 – 4.2 (2.9)	4.0 – 5.2 (4.4)	3.9 – 5.8 (4.9)	5.1 – 6.5 (6.0)	5.3 – 6.1 (5.9)	2.9 – 5.1 (3.9)	
FSL R9-5515	2.0 ±1.0	2.3 ±1.2	3.9 ±0.4	5.8 ±1.9	6.4 ±1.1[2]	6.1 ±0.0[3]	4.5 ±0.2	4.4
FSL R9-5517	2.4 ±0.7	4.2 ±1.3[1]	5.2 ±0.3	3.9 ±0.3	5.4 ±1.7[1]	6.0 ±0.1[3]	5.1 ±0.7	4.6
FSL R9-5258	3.9 ±0.3	4.2 ±1.6[1]	4.1 ±1.5[1]	5.2 ±1.2	5.1 ±1.4[1]	6.1 ±0.0[2]	2.9 ±0.4	4.5
FSL R9-5516	2.7 ±0.8	2.5 ±1.4	4.0 ±2.4	4.2 ±0.3	6.5 ±1.1[2]	6.0 ±0.3[2]	3.4 ±1.1	4.2
FSL R9-5271	3.4 ±1.6	1.5 ±0.6	4.6 ±0.3	5.3 ±0.5	6.5 ±0.1[2]	5.3 ±0.9[2]	3.4 ±0.3	4.3
FSL R9-4077 [b]	5.2 ±1.3	2.4 ±1.6	4.7 ±0.3	5.8 ±1.1	5.3 ±1.6[1]	5.8 ±0.6[2]	2.8 ±0.8	4.6

[a] Numbers shown represent the arithmetic mean of three trials, reported as log reduction ±SD; numbers shown in square brackets indicate the number of trials that yielded bacterial numbers below detection limit after PAA exposure (absence of square brackets indicates that all three replicates yielded data above the detection limit). For rows that list one of the three bacterial groups (e.g., *Listeria monocytogenes*), range of log reduction across all strains tested (excluding surrogates) are shown, arithmetic means for all strains (excluding surrogates) are shown in round parenthesis.

[b] Surrogate organisms are marked with a b superscript
